# Decreases in polyunsaturated fatty acid content improve heat stress tolerance during flowering and silicle development in pennycress (*Thlaspi arvense* L.)

**DOI:** 10.3389/fpls.2026.1811992

**Published:** 2026-05-05

**Authors:** Nikhil S. Jaikumar, Matthew G. Garneau, Brice A. Jarvis, Mary E. Phippen, Winthrop B. Phippen, Philip D. Bates, John C. Sedbrook

**Affiliations:** 1 School of Biological Sciences, Illinois State University, Normal, IL, United States; 2 Institute of Biological Chemistry, Washington State University, Pullman, WA, United States; 3 School of Agriculture, Western Illinois University, Macomb, IL, United States

**Keywords:** climate, FAD2, lipid, oilseed, peroxidation, pollen, PUFA, ROD1

## Abstract

**Introduction:**

Pennycress (*Thlaspi arvense* L.) is an emerging intermediate oilseed crop grown in the offseason between primary summer crops to produce three cash crops in two years. Previous efforts to improve seed oil quality produced Clustered Regularly Interspaced Short Palindromic Repeats (CRISPR) genome-edited lines with decreased polyunsaturated fatty acids (PUFAs) levels, through loss of function of the *FATTY ACID DESATURASE 2* (*FAD2*), *REDUCED OLEATE DESATURATION1* (*ROD1*), and *FATTY ACID ELONGASE1* (*FAE1*) genes. While seed oil compositions were previously characterized, it remains unknown how vegetative and reproductive tissue compositions might differ and affect tolerance to high temperature (HT) conditions.

**Methods:**

In four growth chamber experiments, we explored HT tolerance during flowering and silicle development. Plants were subjected to a 34 °C day/28 °C night regime and compared to control plants maintained at 20 °C. Pollen grain viability at a range of temperatures, lipid peroxidation and proline content in leaves and silicles following HT, and seed yield were measured.

**Results:**

Both *fad2* and *rod1* mutant lines had relatively higher pollen viability (71% and 54% respectively) under moderately elevated temperature (28 °C) compared to wild-type controls (37%). They also showed smaller decreases in seed yield (0% and 40% for *fad2* and *rod1* respectively, compared to 61% for wild type), following HT exposure during late flowering and early silicle development. Silicles of *fad2* plants experienced 65% less lipid peroxidation under HT and 55% less buildup of proline, signifying less stress.

**Discussion:**

The differential results of *fad2* and *rod1* are likely due to the role of FAD2 in membranes in all tissues, whereas ROD1 predominantly affects triacylglycerol (TAG) composition in oil-accumulating tissues including pollen. Our results indicate that decreasing PUFAs, through gene editing, can increase heat tolerance in reproductive tissues as an auxiliary benefit accompanying improved seed oil quality.

## Introduction

Climate change has intensified the frequency, severity, and duration of heat waves globally, thereby reducing crop productivity (Barriopedro et al., 2023; Preece et al., 2023; Sun et al., 2023; Rezaei et al., 2023). Winter annual crops (also known as intermediate crops) are particularly susceptible to heat stress in the spring during flowering, pollination and seed pod development (Lohani et al., 2020). Rising temperatures disrupt physicochemical parameters and cellular membranes by altering fatty acid composition, particularly the balance between saturated and unsaturated lipids (Falcone et al., 2004; Upchurch, 2008; Rahnama et al., 2024, Rahnama et al., 2026).

Two mechanisms by which high temperature (hereafter “HT”) impairs reproductive development are increased membrane fluidity and enhanced lipid peroxidation (Rawat et al., 2021; Chen et al., 2026). Peroxidation of polyunsaturated fatty acids (PUFAs) generates reactive compounds such as malondialdehyde (MDA), which damage proteins and nucleic acids (Kishida et al., 1993; Yamauchi et al., 2008; Yun and Surh, 2012; Endo et al., 2013; Alche, 2019; Ma et al., 2019). These deleterious effects may be mitigated by altering lipid saturation. Increased saturation characterized by decreased PUFA content and higher monounsaturated (MUFA) or saturated fatty acid (SFA) levels enhances membrane rigidity (Hayes et al., 2021; Rawat et al., 2021; Sharma et al., 2023; Spivey et al., 2023), whereas decreasing PUFA levels, particularly linolenic acid and other trienoic fatty acids, would limit substrates available for lipid peroxidation.

Intermediate crops provide many ecosystem benefits during the off-season, including reduced soil erosion, reduced nutrients leaching from farm fields, weed suppression, and pollinator provisions, while also providing farmers with extra income thereby incentivizing their integration into existing agricultural systems (Eberle et al., 2015; Johnson et al., 2017; Thomas et al., 2017; Cubins et al., 2019; Griffiths et al., 2023; Gautam et al., 2025). One promising new intermediate crop is pennycress which has been domesticated from field pennycress (*Thlaspi arvense* L.) (Sedbrook et al., 2014; Chopra et al., 2018; Marks et al., 2021; Gautam et al., 2026). Pennycress is a freeze-tolerant, oilseed-producing Brassica related to rapeseed/canola, carinata, and camelina. Pennycress has a relatively small diploid genome (539 Mb) which has facilitated its rapid domestication (Dorn et al., 2015; McGinn et al., 2019; Nunn et al., 2022; Ma et al., 2023; Basnet and Ellison, 2024; Lee et al., 2024). The first commercial pennycress varieties, trade-named CoverCress™, are being planted within corn-soybean rotations in the U.S. Midwest (Phippen et al., 2022; Gautam et al., 2026). At harvest, the seed oil can be used for biofuels production, as an edible oil, and in lubricants, while the seed meal has value as an animal feed and for industrial applications (Vaughn et al., 2005; Moser et al., 2009; Fan et al., 2013; Mousavi-Avval and Shah, 2020; Al Sulaimi et al., 2023; Hartnell et al., 2023).

Over the last decade, pennycress oil quality has been improved by increasing oleic acid levels (18:1) while decreasing the PUFAs linoleic acid (18:2) and linolenic acid (18:3), and/or decreasing erucic acid (22:1) levels (McGinn et al., 2019; Chopra et al., 2019, Chopra et al., 2020; Jarvis et al., 2021). Wild-type pennycress oil contains about 35% erucic acid and moderate amounts of linoleic acid and linolenic acid (Altendorf et al., 2019). Linoleic acid and linolenic acid, although nutritious, are prone to oxidation resulting in reduced shelf life of the oil and its instability at high temperatures (Warner and Fehr, 2008). Erucic acid as a foodstuff is considered cardiotoxic by regulatory authorities in the United States and European Union (Galanty et al., 2023; Mohanty et al., 2025), and as a biodiesel feedstock causes undesirable increases in viscosity at low temperature (Moser, 2012). Efforts to reduce PUFAs in pennycress oil have targeted the *FATTY ACID DESATURASE 2* (*FAD2*) and *REDUCED OLEATE DESATURASE 1* genes (*ROD1*; Jarvis et al., 2021), while efforts to eliminate erucic acid have targeted *FATTY ACID ELONGASE 1* (*FAE1*; McGinn et al., 2019; Rasoul et al., 2025).

FAD2 is a membrane-bound fatty acid desaturase in the endoplasmic reticulum, which adds a double bond to a phosphatidylcholine-linked oleic acid chain (PC-18:1), to produce PC-18:2 (Hernandez et al., 2009; Dar et al., 2017). 18:2 can then be further converted to linolenic acid (18:3) by FAD3 in the endoplasmic reticulum (ER), or FAD7/FAD8 in the plastid. *ROD1* is a more recently discovered gene (Lu et al., 2009; Bates et al., 2012; Hu et al., 2012) encoding a phosphatidylcholine: diacylglycerol cholinephosphotransferase (PDCT) which transfers phosphocholine from phosphatidylcholine (PC) to diacylglycerol (DAG: Bates et al., *ibid.*) thereby generating new molecular species of PC and DAG. PDCT equilibrates the DAG moiety between the PC and DAG pools, allowing more 18:1-containing DAG to enter PC for desaturation, and more 18:2/18:3-containing DAG to exit PC and be used as a substrate for synthesis of triacylglycerol (TAG; Lu et al., *ibid.*; Bai et al., 2020). TAG stores fatty acids in lipid droplets within seed embryos, reproductive tissues particularly pollen, and vegetative tissues (Guzha et al., 2024).

As the major delta12 desaturase in the ER the *fad2* mutation has a strong effect on both membrane lipid and TAG fatty acid composition in all tissues, whereas PDCT is involved in the transfer of PUFA from PC to other lipids, predominantly TAG and thus has less effect on membrane lipid composition (Bates et al., 2012; Bates and Shockey, 2025). FAE1, meanwhile, functions in a separate pathway to elongate oleic acid (18:1) bound to Coenzyme A stepwise to 20:1 and 22:1 (Wu et al., 2008). FAE1 is expressed in developing seed tissue and predominantly affects seed oil composition. Jarvis et al. (2021) successfully created loss-of-function mutations in pennycress *FAD2* and *ROD1*, increasing 18:1 levels to 35% of seed TAG in the first case and 22% in the second with concomitant reductions in 18:2 and 18:3. Combining *fad2* and *rod1* loss-of-function mutations with the previously created *fae1* mutation (McGinn et al., 2019) increased oleic acid content even further (Jarvis et al., *ibid.).* These results parallel recent successful efforts to knock out *FAD2* in soybean (Wu et al., 2020a; Rathod et al., 2026), camelina (Lee et al., 2021; Van Belle et al., 2025), cardoon (Cappetta et al., 2022), peanut (Rajyaguru and Tomar, 2024; Shi et al., 2025), and canola (Okuzaki et al., 2018; Wang et al., 2025). They also complement recent work with *ROD1* knockouts in *Arabidopsis* (Lu et al., 2009; Wickramarathna et al., 2015) and soybean (Li et al, 2023), and with combined *fad2 fae1* knockouts in canola (Shi et al., 2022) and field cress (Sandgrind et al., 2023).

The development of “high oleic” lines makes pennycress more attractive as an edible oil or biofuel feedstock due to improved oxidative stability. It also raises the question of how changes to lipid fatty acid composition might affect tolerance to HT. Across and within species, HT is often associated with decreased PUFA content or decreased desaturase expression, including in oilseeds such as safflower (Rahnama et al., 2024), sunflower (Rahnama et al., 2026), peanut (Zoong Lwe et al., 2020), soybean (Narayanan et al., 2020), carinata (Zoong Lwe et al., 2021) and camelina (Nastase et al., 2026). These observational studies of the role of lipid unsaturation in heat tolerance are corroborated by knockout studies. CRISPR/Cas9 knockout of stearic acid desaturase in *Pinellia ternate* led to decreased monounsaturated fatty acids (MUFAs) and PUFAs content and correspondingly improved heat tolerance (Zhang et al., 2021). In *Arabidopsis*, knockout of 18:3 synthesis (Murakami et al., 2000; Larkindale et al., 2005; Yamauchi et al., 2008) or 18:2 synthesis (Wallis and Browse, 2002) in photosynthetic tissues conferred improved thermotolerance.

Reproductive stages are often especially susceptible to heat injury, even in plants that are otherwise heat tolerant (e.g. Ahmed et al., 1992; Djanaguiraman et al., 2018a). Pollen contains relatively high levels of TAG as compared to vegetative tissues (Li-Beisson et al., 2013; Bai et al., 2023), and it is believed that TAG turnover provides fatty acid and DAG substrates for rapid membrane lipid synthesis (Ischebeck, 2016) to extend its pollen tube to the ovule. Therefore, pollen viability under heat stress can be highly sensitive to changes in lipid composition (Krawczyk et al., 2022). Tobacco (Krawczyk et al., 2022) and peanut (Zoong Lwe et al., 2020) respond to high temperatures by shifting towards greater lipid saturation in anthers. In other species, both *FAD2* (Dar et al., 2017; Liu et al., 2019; Zhao et al., 2022) and *ROD1* (Lu et al., 2009; Brown et al., 2012) are expressed in flowers, and therefore *rod1* and *fad2* knockout mutations have the potential to affect lipid composition and thereby heat tolerance in pennycress anthers and pollen. Based on the function of each gene it is expected that *fad2* would affect the fatty acid composition of both membranes and TAG, whereas the *rod1* mutation would predominantly affect TAG fatty acid composition. To the best of our knowledge, no previous study has specifically explored the effects of *fad2* or *rod1* knockout on reproductive performance at HT.

**Figure 1:**
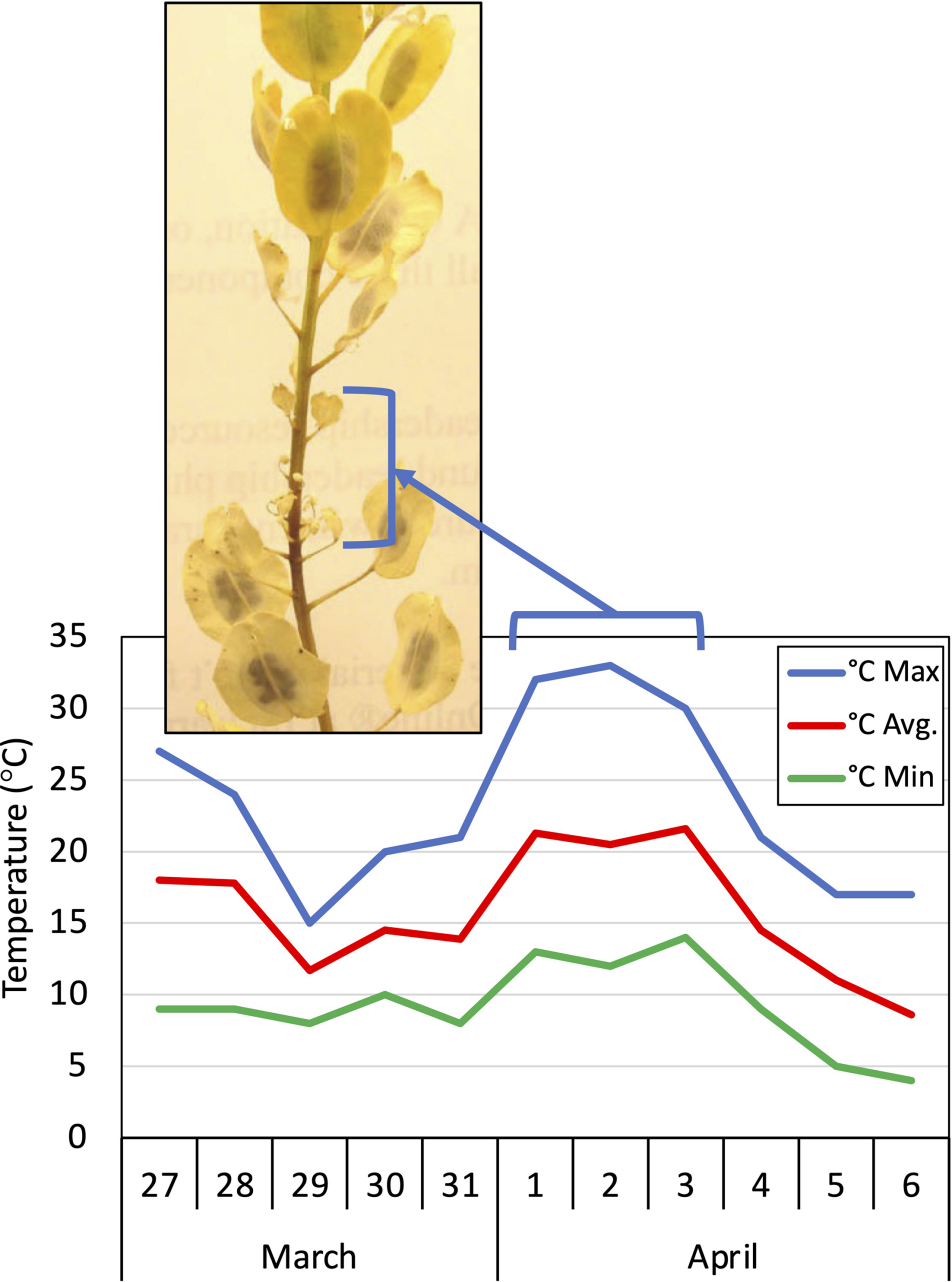
Inflorescence from a field-growing pennycress plant exhibiting aborted seed pods due to temperatures reaching 32 °C (~90°F). Blue brackets delineate the zone of aborted seed pods (sterility) that occurred when the related flowers were exposed to 32 °C temperatures as shown in the March-April temperature history graph for Macomb, Illinois USA. Note that when temperatures returned to below 32 °C (April 4th) the flowers returned to being fertile thereby producing seed-filled pods (blue bracket).

Pennycress is a cold tolerant species, inhabiting temperate and subarctic regions on all continents except Antarctica, with documented sightings as far north as 67°N in Finland and 66°N in Canada (https://www.inaturalist.org/observations?place_id=any&subview=map&taxon_id=79358). While pennycress also inhabits lower latitudes including the southern U.S., plants visibly suffer flower abortion and reduced seed yield when temperatures surpass 30 °C during flowering (**Figure 1**). Increasing heat tolerance while retaining adequate cold tolerance is therefore an important goal in efforts to better adapt domesticated pennycress for heat waves, which are becoming more frequent and severe with climate change (Fischer and Knutti, 2015; Preece et al., 2023). The exact relationship between temperature and pollen viability in pennycress has not been studied in detail; e.g. thresholds for 50% viability loss are unknown. In addition, it is unknown whether pollen viability or pistil fertility is more strongly affected by HT in pennycress. While pollen is commonly considered more heat-susceptible than pistils (Santiago and Sharkey, 2019), some species show the opposite pattern (e.g. Djanaguiraman et al., 2018b). In this study, we seek to address these knowledge gaps, by documentation of how gene editing for low-PUFA seed oil affects the fatty acid composition of reproductive tissues and the response of pennycress to elevated temperature during flowering.

## Materials and methods

### Selection of genotypes for heat stress experiments

Creation of these lines was described in Jarvis et al. (2021), involving CRISPR-genome editing of a pennycress “Spring 32-10” inbred line (also sometimes referred to as “Spring 32” in this manuscript; McGinn et al., 2019). Spring 32–10 is derived from 10 generations of single seed descent from seeds collected near Bozeman, Montana and is currently used as the reference spring-type genotype (Nunn et al., 2022). Experiment 1 involved one *rod1* loss-of-function mutant (+T insertion), two *fad2* loss-of-function mutants (with a +A insertion and a -29 base pair deletion, respectively), one *fae1* loss-of-function mutant (-4 base pair deletion), and two double mutants (a *rod1 fae1* line with a +T insertion and -4 base pair deletion in the two genes, respectively, and a *fad2 fae1* line with a +A insertion and -4 base pair deletion, respectively). The two *fad2* lines will henceforth be referred to as *fad2–4* and *fad2–6* respectively. Experiment 2 involved the same *rod1*, *fae1*, *fad2*, *rod1 fae1*, and *fad2 fae1* lines as in the previous experiment, but also included a distinct *fad2 fae1* allele combination (-2 bp deletion in *fad2* and 4 bp deletion in *fae1*). The (+A, -4) and (-2, -4) lines will henceforth in the text be referred to as *fad2–4 fae1*, and *fad2–5 fae1*, respectively. All mutant lines were generated through CRISPR-Cas9 gene editing as previously described (Jarvis et al., 2021). PCR analysis indicated that *rod1, fae1, fad2* and *fad2 fae1* lines were all free of the Cas9 gene, while the *rod1 fae1* line still contained CRISPR-Cas9 construct. All lines were confirmed to be homozygous for the mutations in *rod1, fae1*, or *fad2* respectively. These analyses were done by amplifying the relevant gene, running the PCR products on an agarose gel, staining with ethidium bromide, isolating and purifying the amplified DNA using a GeneJet Gel Extraction Kit (Thermo Scientific: Waltham, Massachusetts), and Sanger sequencing the resulting template. Sequencing was done by Eurofins Genomics (Louisville, Kentucky).

### Seed planting and transplanting in heat stress experiment

Experiments 1 and 2 were done to assay pollen viability, biochemical stress indicators and seed yield. Experiments 3 and 4 were done to assay lipid composition analysis either in whole flowers or in pollen-enriched floral extracts, respectively, as well as subsequent seed yield. For all experiments, plating and transplanting were done as follows. Seeds were surface sterilized using a 95% ethanol rinse followed by a 20 min incubation in sterilization solution consisting of 0.01% SDS and 30% bleach. Seeds were rinsed four times with sterile water, plated on 0.8% agar with half-strength Murashige and Skoog salts and 0.6 mg mL^-1^ MES in Parafilm-sealed petri dishes, and incubated in a Percival Scientific CU-36L5 incubator (Percival Scientific: Perry, Iowa). Seed incubation conditions were 16-hour light/8-hour dark photoperiod, 150 – 200 µmol m^-2^ s^-1^ light, and 22 °C. Once seedlings had reached appropriate size for transplanting (approximately 12 days for *fad2* and *fad2 fae1* lines, 7 days for other lines), they were moved into a cold room at 4 °C to vernalize for 21 days. The purpose of this step was to mimic conditions that pennycress would experience in the field. Following vernalization, seedlings were transplanted into 500 mL pots filled with a previously sterilized mixture of 50% Miracle-Gro Moisture Control Potting Mix (Miracle-Gro: Marysville, Ohio) and 50% ProMix BX Mycorrhizal (ProMix: Winterthur, Switzerland) with 0.03 g pot^-1^ of the insecticide Marathon and 1.4 g pot^-1^ of Actinovate SP biological fungicide with *Streptomyces lydicus* (Novozymes: Bagsvaerd, Denmark). Half of these pots were in trays dedicated for the HT treatment, and half were treated as controls which would not be heat-treated. Due to differential germination rates at the seedling stage, sample size varied somewhat: from 8–22 with a mean of *n* = 11 in Experiment 1, from 8–18 with a mean of *n* = 12 in Experiment 2, and from 32–40 with a mean of 37 in Experiment 4. Experiment 3 had *n* = 12 total plants per treatment x mutation combination, but because of slight variation between plants in the number of flowers ready for collection on day 7 of the HT treatment, only between *n* = 5 and *n* = 10 with an average of 8 were chosen for flower collection. Trays were placed in a CMP 3244 controlled-environment growth chamber (Conviron: Winnipeg, Canada) and covered with plastic trays for 2 days, after which the plastic was removed and they were grown until shortly before flowering, with watering on alternate days. Growth conditions comprised 20 °C constant temperature, 16-hour light/8-hour dark photoperiod, and light levels (at the soil level) of approximately 185 µmol m^-2^ s^-1^. Plants were treated with Safer^®^ brand sulfur-based fungicide spray as needed during the first week after transplanting (Woodstream Corporation: Lancaster, Pennsylvania). No additional fertilizer or pesticide was applied.

### Protocol for high temperature treatment

In Experiments 1-2, at 25 days after transplanting, shortly before flowering, the “control” plants were left as they were, while plants designated for HT were moved into a LED 41L2 controlled-environment growth chamber (Geneva Scientific: Williams Bay, Wisconsin). Conditions here involved fluctuating temperatures with a daytime high (held for 7 hours) at 20 °C, overnight lows (for 7 hours) of 14 °C, and gradual transitions in between with a rate of change of 1 °C per hour. Photoperiod was 16-hour light/8-hour dark, light levels were 170 - 180 µmol m^-2^ s^-1^ and relative humidity was 55%. After 4 days of acclimating to the chamber, the heat stress treatment was begun. Temperatures were raised by 3 °C the first day (e.g. the 7-hour daytime high was now at 23 °C and the nighttime low at 17 °C, with the same day-night differential and the same gradual transition process). On the second day the temperature was raised by another 3 °C to 26 °C, and on successive days the temperature was raised by a further 2 °C per day, to mimic gradual increases in temperature that might be observed under field conditions and to provide a range of temperatures at which to observe pollen viability, up to a peak of 34 °C day/28 °C night. This “peak” temperature stress regime was held for 9 days, at which time the temperature was gradually ramped down again (with reductions of 2 °C per day for the next four days and 3 °C for the last two). Once the heat stress had ended, plants were allowed to acclimate for 4 days before being switched back to the original growth chamber. Not all of the analyses could be done on all plants, due either to slight variations in growth stage or to insufficient quantity of tissue to sample. For pollen viability analyses there were an average of *n* = 7 replicates per treatment x line combination, with a maximum of *n* = 13 and a minimum of *n* = 4 (for *rod1 fae1* in Experiment 1 and for *rod1* in Experiment 2). For seed yield, all plants were assayed, thus there were from 8–22 plants in Experiment 1 (mean *n* = 11) and between 8–18 plants in Experiment 2 (mean *n* = 12). For TBARS analyses, there were between 5–16 replicates in Experiment 1 (mean *n* = 7) and between 6–9 replicates in Experiment 2 (mean *n* = 7). For proline analyses there were 6–11 replicates in Experiment 1 (mean *n* = 8) and 6–10 replicates in Experiment 2 (mean *n* = 7). For peroxide analyses there were 7–8 replicates in Experiment 1 and 6–8 in Experiment 2 (mean *n* = 7).

Experiments 3–4 followed a slightly different procedure, as here the goal was to measure flowers that had developed completely under a single daytime high temperature. Rather than the gradual ramp-up in temperature, a single extreme HT regime was imposed for 18 days beginning just before floral emergence. Plants in the HT treatment were moved into the same HT growth chamber as described above at 22 days after transplanting. After 4 days of acclimation, shortly before floral emergence, the HT chamber was set to 34 °C day/28 °C night, with humidity and light levels as specified for Experiments 1-2. Following the 18-day HT treatment, by which time flowering was mostly complete, plants were moved back to the control chamber at 20 °C. In all the experiments, plants had senesced by approximately 9–10 weeks after flowering: seeds and biomass were then harvested and weighed. For seed yield and biomass estimates, sample size was *n* = 12 in Experiment 3, and varied from *n* = 32 to *n* = 40 with an average of *n* = 38 per treatment x mutation combination in Experiment 4. Floral lipid composition and pollen lipid composition were estimated in Experiments 3 and 4 respectively, with between 6–10 biological replicates (mean *n* = 8).

### Whole flower and pollen fatty acid composition in heat stress experiment

Whole unopened flowers and pollen were collected from experiments 3 and 4, respectively, in both cases after 7 full days of 34 C day / 28 C night treatment. Each replicate was drawn from 1 plant in Experiment 3 and 3–4 plants in Experiment 4. Flowers were selected immediately prior to opening. Whole flowers were immediately frozen into liquid N_2_ and stored at -80 °C until analysis. Pollen was freshly collected from ≈25 (3–4 per biological replicate) individual plants by first pinching the flower at the base of the peduncle and placing them in a 2.0 mL pre-chilled tube, on ice, containing 1.75 mL distilled water. Flowers were then vortexed and centrifuged at 1000 x *g* for 1 min to collect the majority of the floral tissue. Next, samples were centrifuged at 10,000 x *g* for 2 min to pellet pollen, the supernatant removed, and the resulting pellet frozen in liquid N_2_ and stored at -80 °C until analysis. Flowers were weighed (~20 mg per sample), then transferred to 8 mL glass tubes with PTFE lined caps. Pollen was transferred to 8 mL glass tubes with 3 successive washes of 0.5 mL 5% H_2_SO_4_ in methanol. Whole tissue transmethylation to fatty acid methyl esters (FAME) was performed on pollen and floral tissue with fatty acid amount and composition determined by gas chromatography with flame ionization detection (GC-FID). The transmethylation followed a method modified from whole seed transmethylation (Garneau et al., 2025) as follows. To each tube containing ~20 mg flowers, 1 mL of 5% H_2_SO_4_ in methanol (omitted for pollen, see above), then 40 µg Tripentadecanoin (15:0-TAG) internal standard in 200 µL toluene was added, samples capped, and heated at 85 °C for 90 min. After cooling, 0.5 mL of hexane and 1.5 mL 0.88% KCl were added, vortexed, and centrifuged at 2500 x g for 5 min to separate the phases. 5 µl of hexane fraction was injected into an Agilent 7890 GC-FID with Agilent DB HEAVYWAX UI column (30 m length, 0.25 mm inner diameter, 0.25 µm film thickness). Samples were run at a split ratio of 1:10 for floral and 1:1 for pollen samples. Both the GC injector and flame ionization detector were held at 255 °C with a hydrogen flow of 1.25 mL min^-1^. The oven program followed a 2-step ramp that started at 170 °C and increased at 20 °C min^-1^ to 200 °C, then at 7.5 °C min^-1^ to 260 °C, followed by a 3 min hold. Identification of fatty acids was based on retention time to GLC421A and GLC490 FAME standards (Nu-Chek Prep, Inc.). Peak area was analyzed using Openlab CDS ChemStation edition (C.01.09) and compared to 15:0 internal standard.

### Seed fatty acid composition in heat stress experiment

In Experiment 1 only, total seed lipids were transmethylated directly from intact seeds to produce fatty acid methyl esters (FAMEs) for gas chromatography (GC) analysis. A Mettler Toledo analytical balance (Mettler Toledo: Columbus, Ohio) was used to weigh 100 mg of seed from each plant into 20 mL glass scintillation vials. To each sample, 5mL of 0.25 M sodium methoxide in methanol was added, and seeds were homogenized with a Ingenieurburo CAT X 120 homogenizer. The vials were capped and incubated at 60 °C for 30 min, then cooled to room temperature. Subsequently, 2 mL of saturated NaCl and 5 mL of hexane were added. After shaking, the upper layer containing FAMES was collected for GC analysis.

FAMEs were analyzed using an Agilent 6890 gas chromatograph (Agilent: Santa Clara, California) with a 7683 autosampler and flame ionization detector (FID). A Supelco 2380 30m x 0.25mm x 0.2µm film thickness column (Supelco: Bellefonte, Pennsylvania) was used. Ultra-high purity helium was the carrier gas with a constant pressure of 20.00 psi. A 1 µL injection was used with a split ratio of 50:1. Split flow and total flow were 58.3 mL min^-1^ and 62.2 mL min^-1^. For the detector, ultra-high purity hydrogen and air were used in the FID with flow rates of 40.0 mL min^-1^ and 450.0 mL min^-1^ respectively. The injector and detector temperatures were set at 265 °C and 250 °C respectively. The oven temperature was set initially at 170 °C and ramped at 4 °C min^-1^ to 190 °C then ramped at 30 °C min^-1^ to 265 °C and held for 2.5 min. NuChek standard 17A and Supelco 37 component FAME standard were run to match retention times. Data was collected and the area percents for the FAMEs were exported into an Excel file using Agilent OpenLab CDS ChemStation Edition revision C.01.10 software.

### Assay for pollen viability under heat stress

Pollen viability was tested using the TTC (triphenyl tetrazolium chloride) staining method (Zheng et al., 2021; Wu et al., 2020b). Pollen was collected each day during the 1 week ramp-up portion of the HT treatment (starting with the day immediately before the temperature ramp-up started, at 20 °C, and subsequently at higher temperature on each successive day). Pollen collection took place at the end of the 7-hour peak heat stress, at the equivalent of 13 hours after dawn. Approximately 6–8 flowers per plant were collected and smeared on a clean glass microscope slide to dislodge anthers and free pollen from the flower. Anthers and pollen were treated with 600 µL of TTC solution (100 mM phosphate buffer at pH = 7.5, 1.5% sucrose, 1% TTC) and covered with a cover slip which was sealed with nail polish around the edges. Slides were incubated for 2 hours at 25 °C and an additional 2 hours at 37 °C and were then examined using a Leitz DM-RBE compound microscope (Leica: Wetzlar, Germany). Approximately 200 pollen grains per plant were observed and were rated either “viable” if they stained red or “nonviable” if they were unstained and pale yellow or clear. Viability was assessed as the percentage of viable (red stained) pollen grains out of total grains.

### Lipid peroxidation levels under heat stress

Lipid peroxidation was estimated on leaf and silicle tissue samples in Experiment 1 and 2. Lipid hydroperoxides are formed from unsaturated lipids, especially PUFAs, under oxidative stress, particularly at HT (Kishida et al., 1993; Yamauchi et al., 2008; Yun and Surh, 2012; Endo et al., 2013) and provide information on a mechanism by which changes in saturation might modulate effects of HT. Specifically, we quantified thiobarbituric acid reactive substances (TBARS), following Skrypnik et al. (2021). TBARS is commonly used as a measure of lipid peroxidation, since malondialdehyde (MDA), a common byproduct of lipid peroxidation, reacts with thiobarbituric acid (TBA) to form a yellow adduct whose absorbance is measured. MDA is toxic in its own right and can damage macromolecules, in addition to its significance as a stress indicator. Leaf and silicle samples were collected from HT plants on the last day of the peak temperature treatment in both Experiments 1 and 2 (i.e. the 9^th^ day at 34 °C), and from the control, non-stressed plants the following day, at the equivalent of 12–14 hours after dawn. The silicle samples comprised silicles derived from flowers which had been fertilized at 28 - 30 °C (i.e. which had experienced the entire silicle-filling stage at HT). Leaf samples comprised the 4^th^ leaf starting from the youngest fully expanded leaf at the top of the plant. Samples were collected on dry ice and stored at -80 °C before analysis. Samples were ground using a Retsch TissueLyser II ball mill (Qiagen: Hilden, Germany) and extracted for 30 min at 4 °C (with agitation) in 100 mM phosphate buffer (pH = 7.5) with 75 mM Na_2_SO_4_, 0.55% Triton X-100, 5 mM EDTA, 1 mM EGTA and 8 mM DTT. 100 µL of extract was added to 900 µL of solution including 1.25% trichloroacetic acid, 37 mM TBA, 2.8 M acetic acid and 0.7 mM sodium acetate. The solution was incubated for 120 min at 95 °C, immediately cooled to 4 °C, and centrifuged for 10 min at 2000 g and 4 °C. Absorbance of the TBA - TBARS adduct was read at 532 nm (after subtracting nonspecific absorbance at 600 nm), and compared to a standard curve of 0 - 50 µM MDA in 100 mM phosphate buffer.

### Proline levels under heat stress

Free proline levels were measured in leaf and silicle tissues collected on the last day of the 9-day HT treatment. Proline serves as a protectant under various abiotic stresses, and can serve as a useful indicator of stress as well as a defense compound (Schlafleitner et al., 2007; Spormann et al., 2023). Proline was measured in the same set of sample extracts collected for MDA analysis, following Chen and Zhang (2016) with modifications. Substrate solution was prepared with 1.25% ninhydrin in a 80:20 mix of acetic acid:water. Approximately 167 µL of sample extract was combined with 167 µL of 9% 5-sulfosalicylic acid and 667 µL substrate solution: these were boiled for 60 min, immediately cooled to 4 °C, and centrifuged for 10 min at 13–000 *g*. Absorbance of the proline-ninhydrin derivative was read at 510 nm and compared to a standard curve (0–8 mM proline in 100 mM phosphate buffer, pH = 7.5).

### Total hydroperoxide levels

As an additional indicator of overall reactive oxygen species buildup, the ferrous oxidation/xylenol (FOX) assay was performed to measure hydrogen peroxide content in leaf and silicle extracts collected from heat-stressed and non-stressed plants. Peroxides are one of the most important reactive oxygen species and an indicator of oxidative stress. The assay was done on a separate set of samples from the malondialdehyde and proline assays, following Pal and Kar (2019). Metabolites were extracted in 500 μL of 100 mM potassium phosphate buffer, pH = 7.5, with 1% polyvinylpolypyrrolidone and 5% trichloroacetic acid, for 20 min at 4 °C with agitation: these were centrifuged for 10 min at 4 °C and 12000 rpm. Before the assay, the FOX reagent was prepared by mixing 1-part ferrous ammonium sulfate reagent (25 mM FeSO_4_, 25 mM (NH_4_)_2_SO_4_, 2.5 mM H_2_SO_4_) with 100 parts xylenol orange dye (100 mM sorbitol, 125 µM xylenol orange). 20 µL of sample extract was mixed thoroughly with 200 µL of FOX reagent, incubated for 20 min at room temperature, and absorbance was read at 595 nm in a plate reader. Absorbance was compared to a hydrogen peroxide standard curve (1 - 120 µM hydrogen peroxide in 100 mM phosphate buffer, pH = 7.5).

### Heat stress effects on male vs. female fertility

To determine whether there were differential effects of HT on male and female fertility, reciprocal crosses were performed on wild-type (Spring 32) plants. The goal was to assess fertility of crosses in which either only the male parent, only the female parent, or neither, had been exposed to a transient, 6-hour HT treatment. Spring 32 seeds were sterilized, plated, allowed to form seedlings and then transplanted into pots, following the same protocol as in Experiments 1 and 2. 20 of the 40 total plants were set aside to be “heat stressed”, and 20 were designated to be “non-stressed”. Plants were grown in a CMP 3244 controlled-environment growth chamber with a 16-hour light/8-hour dark photoperiod, and constant 21 °C temperatures, up until 2 days after the first flowers emerged. At this point the reciprocal cross experiment was initiated and was carried out over 4 days. Each day, at 8:30 am, flowers ready for crossing (with white petals visible but still unopened) were identified. Flowers designated to serve as the female parent in the cross (on both heat-stressed and non-stressed plants) were labeled and all anthers carefully removed. Approximately 3–7 flowers were selected, per plant, to serve as “female parents”. Plants designated for HT were then introduced into a LED 41L2 controlled-environment growth chamber (Geneva Scientific: Williams Bay, Wisconsin) maintained at 34 °C with 170 - 200 µmol m^-2^ s^-1^ light and 55% humidity and were kept there for 6 hours. At 4:00 pm plants were removed from the chamber and crosses were performed. Anthers were removed from a flower designated as the “male” parent in the cross and were brushed on the stigma of the “female” parent until pollen could be clearly seen to adhere to the stigma. Plants were then restored to their original chamber at 20 °C. The success or failure of crosses was checked two days later (by determining whether the pistil had developed purple coloration, indicative of a successful cross) and was checked again 10 days later (by determining whether a silicle had formed).

### Seed viability

In Experiment 2, seed collected at harvest (from all of the genotype x stress combinations) was used for seed viability testing. Seeds were surface sterilized and plated as described above (ca. 36 per plate). The plates were then incubated in a LED 41L2 controlled-environment growth chamber (Geneva Scientific: Williams Bay, Wisconsin) Photoperiod was 16-hour light/8-hour dark, light levels were 100 µmol m^-2^ s^-1^, temperature was 20 °C and relative humidity was 55%. Germination was measured from day 3 to day 14. After 14 days plates were scored for total germination.

### Yield

At senescence, plants were harvested, seeds and chaff separated by sieving, and seeds were weighed. In Experiments 3–4 total dry biomass was also weighed. Reproductive allocation was estimated as the ratio of seed weight to total biomass.

### Statistical analyses

Seed and biomass yield, content of each fatty acid in the seed oil, proline content, peroxide content, TBARS content and seed viability were each analyzed by a two-way ANOVA, with “mutation” (considering each of the two mutations to *fad2* separately), “temperature” (HT vs. control), and the interaction of mutation and temperature as explanatory variables. Seed yield, biomass and reproductive allocation in Experiments 3–4 were square root transformed. TBARS content in silicles and, in Experiment 1 only, in leaves, was cube root transformed. Proline content was square root transformed and peroxide was log transformed. All data transformations were done to improve homogeneity of variance, assessed by Hartley’s test. Pollen viability at each temperature was analyzed separately, by a one-way ANOVA with mutation as the explanatory variable. *Post-hoc* comparisons between mutant lines or between mutation x temperature combinations were done using Tukey’s HSD test. Analyses were done in STATA 16.1 (Statacorp: College Station, Texas). Changes in flower and pollen individual fatty acids were analyzed by one-way ANOVA with each genotype x treatment combination treated as an independent group using GraphPad Prism version 10.6.0 for Windows. Significant differences between groups were analyzed using Holm-Šídák multiple comparison test.

### Data presentation

In the Results and Discussion sections below, data is presented separately for the four experiments rather than being pooled. The purpose was to show that trends were consistent across experiments, and that despite variability within and across experiments, mutant lines showed similar relative differences from the wild type in their response to heat stress. Seed yield, for example, was variable within a treatment and between experiments (e.g. non-stressed wild type seed yield had a 27% coefficient of variation in Experiment 1, and a 15% decrease between Experiments 1 and 2) thus we show the two experiments separately to show that the effects of the mutations on the responses to HT were consistent across both experiments.

## Results

We had observed that pennycress plants exposed to heat waves (temperatures attaining or above ~32 °C) while flowering in farm fields and in greenhouses, subsequently produced aborted seed pods (silicles), likely due to heat-induced sterility (**Figure 1**). Those observations prompted us to explore how fatty acid compositional differences might affect pennycress fertility, stress responses and stress damage in reproductive tissues at elevated temperatures.

In two growth chamber experiments (Experiments 1-2), we examined responses to HT imposed during the flowering and silicle development stages, of pennycress lines developed for reduced PUFAs content in seed oil. These had been previously generated through CRISPR/Cas9-induced knockout of *FAD2* and *ROD1*, either alone or in combination with *FAE1*. The HT treatments comprised a 9-day treatment with daytime highs of 34 °C, diurnal temperature variation, and gradual (1 week-long) increases and decreases in temperature before and after the 9-day treatment. These were compared to “control” plants grown at 20 °C throughout. Pollen viability was measured on each day during the week of rising temperatures. Three potential biochemical stress indicators were measured in leaf and silicle tissues at the end of the HT treatment: thiobarbituric acid reactive substances (TBARS) as a proxy for lipid peroxidation, peroxide levels, and proline content. Seed yield and seed lipid composition were measured at harvest. In two subsequent experiments (Experiments 3-4), an 18-day constant HT treatment (34 °C day/28 °C night) was imposed just before flowering. Whole flowers were collected on day 7, for either floral lipid or pollen lipid analysis, and yield and biomass measured at harvest. We predicted that mutations in *FAD2* and *ROD1* would be associated with 1) less fatty acid unsaturation in pollen, flowers, and seeds, 2) greater pollen viability at HT, 3) less lipid peroxidation (i.e. lower TBARS content) following HT, and 4) a smaller reduction in seed yield following HT, compared to wild type.

### Fatty acid compositions of membrane and oil accumulating reproductive tissues of *fad2*, *rod1*, and *fae1* mutant lines

**Figure 2:**
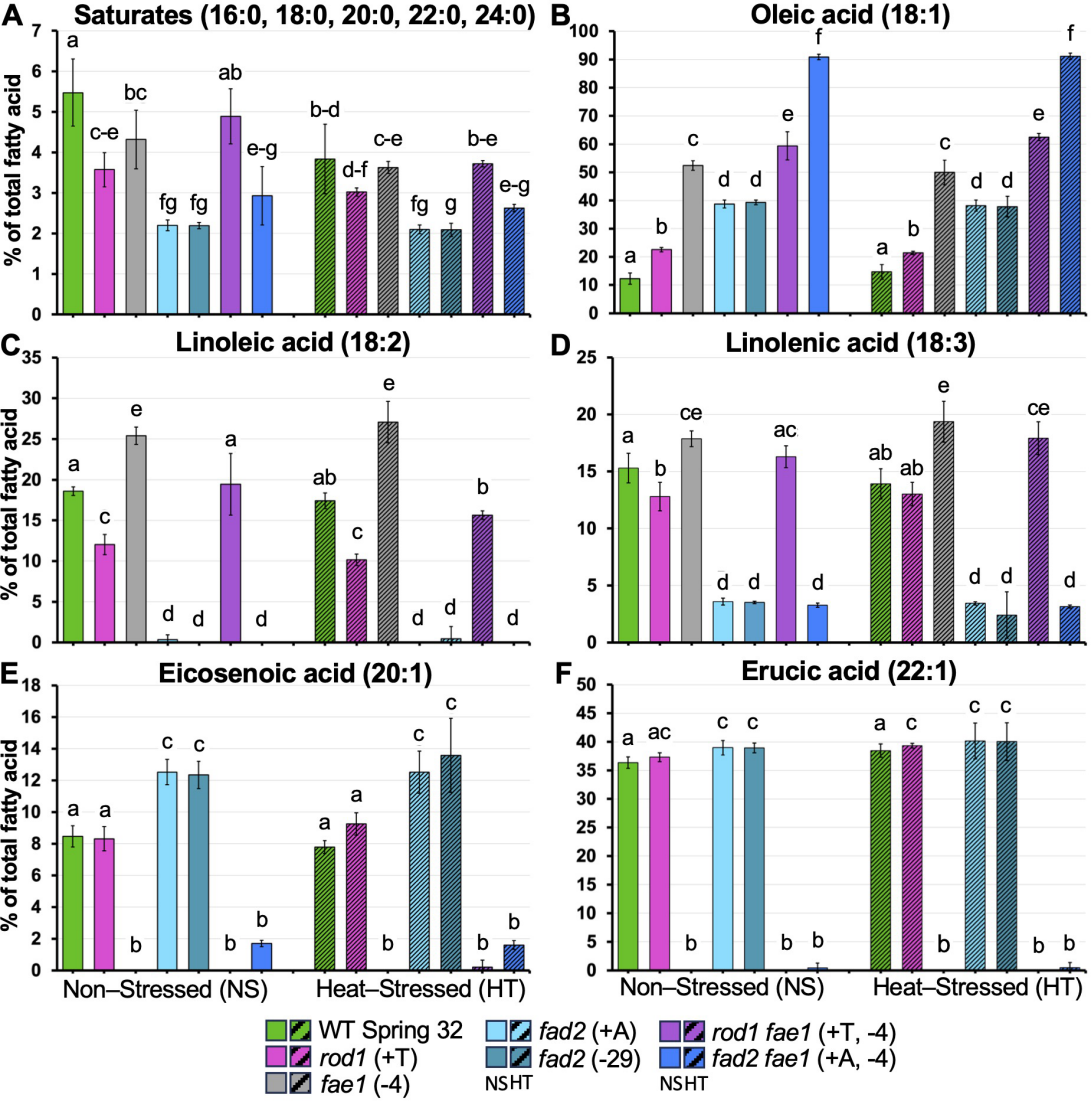
Fatty acid composition of seed TAGs from non-stressed plants (NS; solid columns) versus heat-stressed plants (HT; hashmarked columns). Graphs show mean percents of the total fatty acids (± standard deviation), for **(A)** saturated fatty acids (16:0 through 24:0), **(B)** oleic acid (18:1), **(C)** linoleic acid (18:2), **(D)** linolenic acid (18:3), **(E)** eicosenoic acid (20:1), and **(F)** erucic acid (22:1). Genotypes for all graphs are listed in the color-coded legend at the bottom. Heat stressed plants were grown until flowering at 20 °C; temperatures were then progressively raised by 2 – 3 °C per day up to 34°C, held at 34 °C for 9 days, then reduced by 2 – 3 °C per day back to 20 °C. Non-stressed plants were grown at 20 °C throughout. Means with different letters are statistically significantly different (p < 0.05), as determined by two-way ANOVA followed by Tukey’s test, comparing the HS versus NS treatment for a given mutant or WT. Sample size varied from *n* = 4 to *n* = 20 with an average of *n* = 7.

The *fad2* loss of function in different plant species has been shown to alter fatty acid compositions of both membranes and TAG. By contrast, *rod1* and *fae1* loss of function have primarily been reported as affecting the fatty acid composition of seed TAG: effects on reproductive tissue fatty acid compositions have been little explored. Therefore, we analyzed the fatty acid content of reproductive tissues that vary in membrane lipid versus TAG content including whole unopened flowers (fatty acids mostly in membranes), a pollen enriched flower extract (a TAG rich tissue), and whole mature seeds (predominantly TAG). Since each mutant line was created to enhance seed oil composition, we first analyzed seed fatty acid content in control and HT seeds (parent plants grown at 20 °C and 28-34 °C temperatures, respectively) by GC-FID. Analysis of seed lipid composition confirmed the previously documented phenotypes of seeds from plants grown under minimally stressed conditions (20 - 22 °C; Jarvis et al., 2021). Content of total saturates (sum of 16:0, 18:0, 20:0, 22:0 and 24:0, fatty acid nomenclature # carbons: # double bonds), oleic acid (18:1), linoleic acid (18:2), linolenic acid (18:3), eicosenoic acid (20:1), and erucic acid (22:1) in the seed oil of the fatty acid biosynthetic mutants and wild type are shown in **Figures 2A–F**, in both control and HT plants. Saturated fatty acid (SFA) levels were low in non-stressed wild type (5.1 – 5.9%) and decreased even further under heat stress, to 2.9 – 4.7%. SFA levels were also lower in the *fad2, rod1* and *fae1* lines compared to wild type, but no effects of heat stress were seen in these lines (**Figure 2A**). Levels of oleic acid, linoleic acid, linolenic acid, eicosenoic acid, and erucic acid (**Figures 2B–F**) were consistent with those previously seen in these lines by Jarvis et al. (2021). These changes were mostly maintained under heat stress. Linoleic acid content in the *rod1 fae1* was affected by heat stress (*F*_1,86_ = 8.02, *p* = 0.006) as well as by the stress x mutation interaction (*F*_6,86_ = 5.85, *p* < 0.0001), decreasing from 19% to 16% under heat stress (**Figure 2C**).

**Figure 3:**
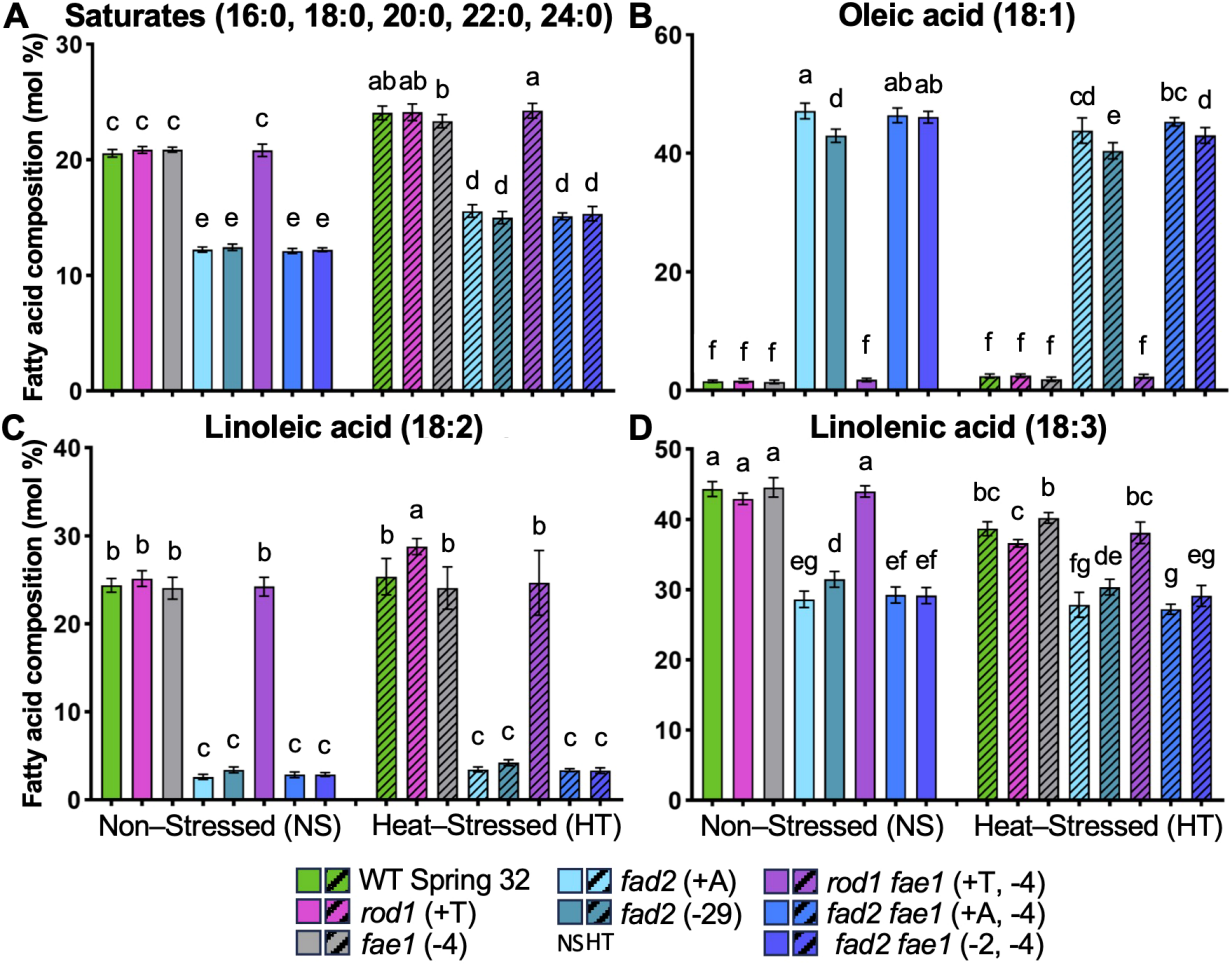
Fatty acid composition of entire flowers from non-stressed plants (NS; solid columns) versus heat-stressed plants (HT; hashmarked columns). Graphs show mole percents of the total fatty acids (± standard deviation), for **(A)** saturated fatty acids (16:0 through 24:0), **(B)** oleic acid (18:1), **(C)** linoleic acid (18:2), and **(D)** linolenic acid (18:3). Heat-stressed plants were grown until flowering at 20 °C: temperatures were then progressively raised by 2 – 3 °C per day up to a 34 °C daytime high, held at 34 °C for 9 days, then reduced by 2 – 3 °C per day back to 20 °C. Non-stressed plants were grown at 20 °C throughout. Statistical differences were determined by ANOVA and the Holm-Šídák multiple comparison test. Significant differences in means are denoted with letters (P ≤ 0.05) between lines, sample size n = 5-10.

In whole flower samples, the total fatty acid composition of non-stressed flowers also reflected expectations for the *fad2*, *rod1*, and *fae1* mutants, in tissues composed predominantly of membranes (**Figure 3**; **Supplementary Figure 1**). Plant membranes have extremely low levels of fatty acids greater than 18 carbons in length, which is consistent with the pennycress flower tissues. Thus, as expected with almost no very long chain fatty acids the *fae1* mutant had no effect on flower fatty acid composition. Likewise, the *rod1* mutant predominantly affects TAG composition, and thus the lack of effect on whole flower fatty acid composition is consistent with its limited role on membrane lipids (**Figure 3**; **Supplementary Figure 1**); However, due to the key role of *fad2* on membrane lipid composition, all lines containing the *fad2* mutation had greatly increased 18:1 (from ~1.5% to ~43 - 47%; **Figure 3B**), and correspondingly decreases in 18:2 (from ~24.4% to ~2.6 - 3.4%; **Figure 3C**) and 18:3 (from ~44.3% to ~28.6 - 31.5%; **Figure 3D**). There were no changes in 16:3 content (**Supplementary Figure 1**), confirming that the changes in flower membrane lipid content are due to endoplasmic reticulum localized eukaryotic pathway FAD2 fatty acid desaturation for both phospholipids and galactolipids, and not due to changes in the prokaryotic pathway FAD6/7/8 fatty acid desaturases (Li-Beisson et al., 2013).

At HT, flower tissues in all lines demonstrated an increase in saturated fatty acids (16:0, 18:0) (**Figure 3A**). The WT, *rod1*, *fae1, and rod1 fae1* mutants had essentially the same fatty acid composition in control conditions, and under HT demonstrated small increases in 18:1 (from ~1.5% to ~2.5%; **Figure 3B**), no change in 18:2 (**Figure 3C**) and slight decreases in 18:3 (from ~44.3% to ~36.6 - 40%; **Figure 3D**). Thus, the changes in flower SFA, MUFA and PUFA compositions for WT, *rod1*, and *fae1* are consistent with the need for decreased membrane fluidity at high temperature, as previously reported in other species. In the *fad2* and *fad2 fae1* backgrounds the increases in saturated fatty acids at HT (from ~12-13% to ~14-16%; **Figure 3A**) were mostly compensated by decreases in the already very high 18:1 content (~43-47% to ~40-44%; **Figure 3B**), with little to no changes in either 18:2 or 18:3 content (**Figures 3C, D**). It is important to note that the PUFA content of the flower *fad2* background under control conditions is already much lower than the WT, *rod1*, and *fae1* backgrounds at HT. Therefore, the lack of change in 18:2/18:3 content of the *fad2* backgrounds at HT likely reflects that fatty acid composition was already better adapted for high temperature.

**Figure 4:**
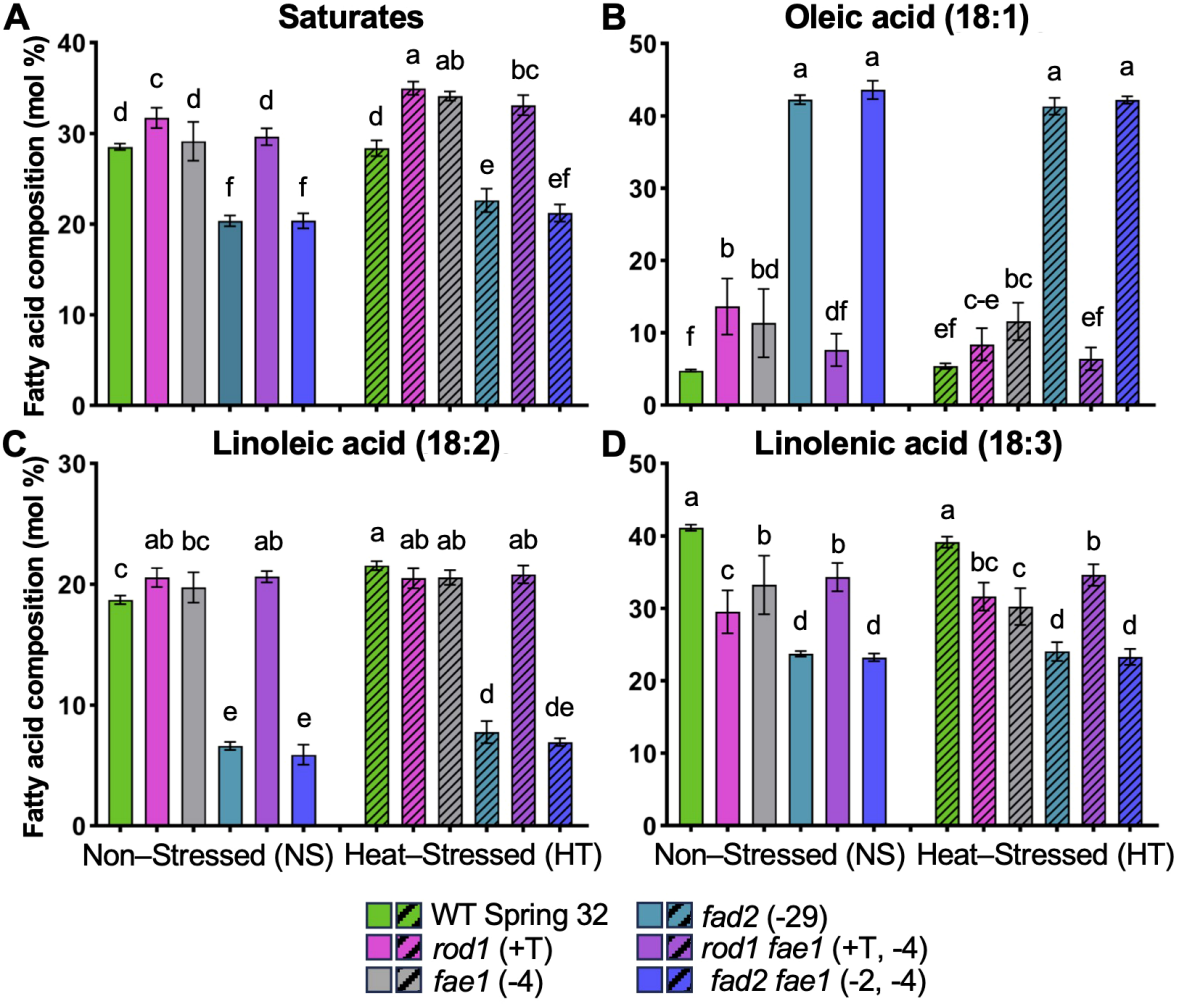
Fatty acid composition of pollen enriched fraction from the flowers of non-stressed plants (NS; solid columns) versus heat-stressed plants (HT; hashmarked columns). Graphs show mole percents of the total fatty acids (± standard deviation), for **(A)** saturated fatty acids (16:0 through 24:0), **(B)** oleic acid (18:1), **(C)** linoleic acid (18:2), and **(D)** linolenic acid (18:3). Heat-stressed plants were grown until flowering at 20 °C: temperatures were then progressively raised by 2 – 3 °C per day up to a 34 °C daytime high, held at 34 °C for 9 days, then reduced by 2 – 3 °C per day back to 20 °C. Non-stressed plants were grown at 20 °C throughout. Statistical differences were determined by ANOVA and the Holm-Šídák multiple comparison test. Significant differences in means are denoted with letters (P ≤.05) between lines, sample size n = 6-10.

Pollen lipids are rich in TAG (but unlike seeds contain very little fatty acids >18-carbons **Supplementary Figure 2**), yet pollen are not predominantly composed of TAG as seeds are. Thus, the changes in fatty acid content in the pollen enriched fraction (**Figure 4**; **Supplementary Figure 2**) due to *fad2* were similar to whole flowers/seeds (**Figures 2**, **3**) which affects both membranes and TAG. The *fae1* mutant had limited effect on pollen lipids apart from a small shift in saturate fatty acid composition (**Figure 4A**). However, it was clear that the *rod1* mutation, that was previously characterized only in seed tissues, also affects lipid composition in pollen (**Figure 4**). Major fatty acid composition changes in the *rod1* pollen enriched fraction included a shift to less unsaturation, with a large decrease in 18:3 (41% to 29%; **Figure 4D**) and corresponding increases in 18:1 (5% to 14%; **Figure 4B**) and saturated fatty acids (28% to 32%; **Figure 4A**). Under high temperature, major changes to *rod1* pollen included an increase in saturates (32% to 35%; **Figure 4A**) and a corresponding decrease in 18:1 (14% to 8%; **Figure 4B**). Thus, *rod1* pollen lipids overall are less unsaturated than WT control, and even more so during heat stress. However, the *rod1* pollen still contains more polyunsaturates than the *fad2* mutant under both non-stressed and heat stressed conditions. Fatty acid composition of the pollen enriched fraction of *rod1 fae1* lines are intermediate between the parent lines and likely result from metabolic changes measured in both (**Figure 4**).

### Pollen viability at moderately elevated temperature

**Figure 5:**
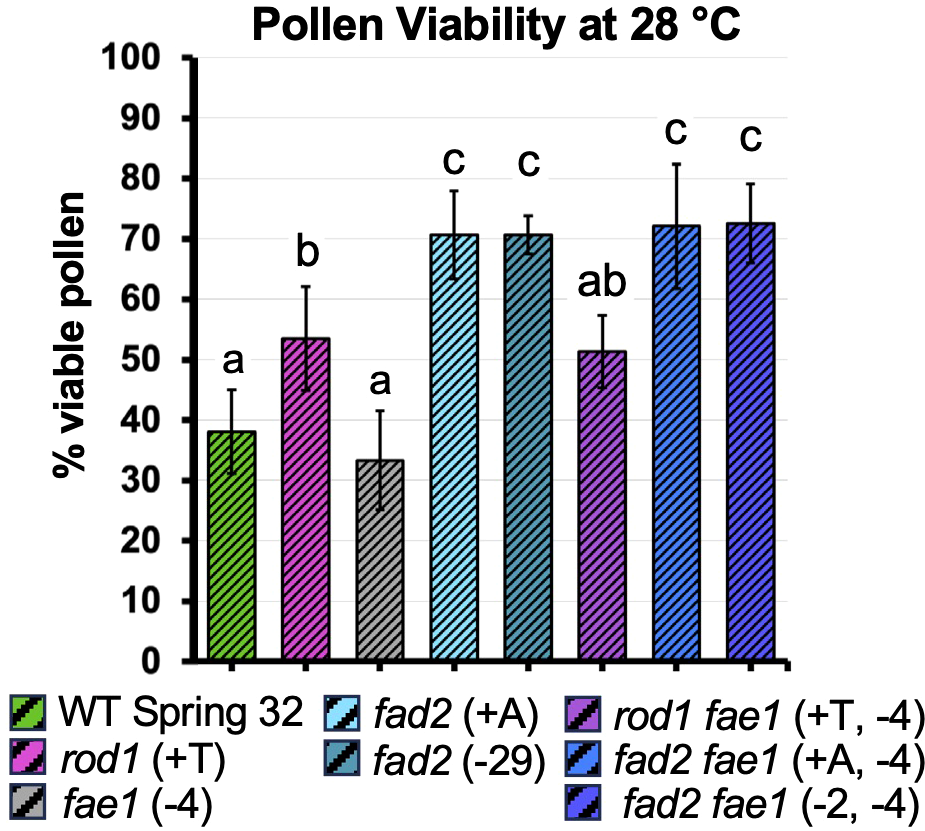
Pollen viability in the flowers of plants exposed to 28 °C. Data from Experiment 2. Flowering plants were exposed to progressively increasing temperatures (increases of 2 – 3 °C in daytime highs), on the days when peak daytime temperatures were set at 28 °C. Columns represent means and bars standard deviations. Statistical differences were measured by ANOVA and the Tukey multiple comparison test. Means with different letters denote significant differences (P ≤ 0.05) between lines, sample size n = 4–11 with an average of n = 7. See **Table 1** for pollen viability percentages at other temperatures.

Both *fad2* and to a lesser extent *rod1* knockout mutations had strong and consistent beneficial effects on pollen viability at moderately high temperatures (in the 26 - 30 °C range), helping to partially ameliorate effects of heat stress. The effects were seen across both Experiment 1 (**Supplementary Table 1****;** the 28 °C data also shown in **Supplementary Figure 3**) and Experiment 2 (**Table 1**; the 28 °C data also shown in **Figure 5**), were stronger for the interrogated *fad2* mutant lines compared to the *rod1* mutant line and were evident from analyses of variance which indicated significant effects of the mutation x temperature interaction. In both experiments, pollen viability in the Spring 32 wild-type control was sharply decreased under elevated temperatures. For example, wild-type pollen viability measured in Experiment 2 dropped from 94 - 100% at 20 °C to 32 - 44% at 28 °C, to only 1 - 13% at 34 °C (*t* = 22.19, *df* = 8, *p* < 0.0001 for the difference between 20 °C and 28 °C: **Table 2**). The decrease in viability with temperature in the *fad2* lines and in the *rod1* line were much more gradual. In Experiment 2, for example, pollen viability at 28 °C was 32 - 44% in the wild-type control versus 68 - 73% in the *fad2–6* line (*t* = 7.95, *p* < 0.0001; **Figure 5**). The *rod1* mutant showed an intermediate level of pollen viability, consistent with its intermediate lipid phenotype (45 - 61%; *t* = 3.55, *p* = 0.018 for the comparison against wild type, and *t* = 4.18, *p* = 0.003 for the comparison against *fad 2- 6*).). Similar effects were seen at 26 °C, where viability (again, in Experiment 2) was 56 - 67% in the wild type compared to 79 - 87% in *fad2–6* plants (*t* = 8.09, *p* < 0.0001), as well as at 30 °C (47 – 57% for *fad2–6* vs. 9 – 15% for wild type: *t* = 14.47, *p* < 0.0001). At 30 °C, similarly to 28 °C, *rod1* viability levels were statistically significantly different from both *fad2* (*t* = 5.32, *p* < 0.0001 for the *rod1* vs. *fad2–6* comparison) and the wild type (*t* = 8.63, *p* < 0.0001) while at 26 °C the *rod1* and *fad2* plants performed similarly. Trends seen in Experiment 1 were similar: e.g. viability at 28 °C was 33 - 40% for the wild type and 68 - 73% in the *fad2–6* plants (*t* = 13.21, *p* < 0.0001; **Supplementary Figure 3**). An alternative way to conceptualize these data is that, in Experiment 2 (for example) the temperature threshold for 50% viability loss was 26 - 27 °C for the wild type, 28 - 29 °C for *rod1* plants, and 30 °C for *fad2* lines.

**Table 1:** Pollen viability in the flowers of plants exposed to different temperatures.

Accession	20 °C	23 °C	26 °C	28 °C	30 °C	32 °C	34 °C
	%						
Spring 32 (WT)	97.9 ± 1.8^a^	92.5 ± 5.7	61.4 ± 6.2^a^	38.0 ± 6.9^a^	12.2 ± 3.4^a^	10.0 ± 4.0^a^	7.0 ± 6.5
*rod1* (+T)	95.7 ± 4.3^a^	92.6 ± 2.6	74.4 ± 4.8^bc^	53.4 ± 8.6^b^	37.2 ± 4.8^b^	17.2 ± 3.0^bc^	10.2 ± 6.9
*fae1* (-4)	97.5 ± 3.3^a^	90.3 ± 6.4	65.7 ± 9.9^ab^	33.3 ± 8.2^a^	16.6 ± 5.3^a^	9.0 ± 2.2^a^	0.5 ± 1.0
*fad2- 4* (+A)	81.5 ± 3.5^b^	91.5 ± 5.0	81.7 ± 3.8^cd^	71.5 ± 7.4^c^	51.4 + 4.9^c^	20.4 ± 2.1^cd^	3.2 ± 5.3
*fad2- 6* (-29)	82.6 ± 3.2^b^	93.9 ± 3.3	82.6 ± 5.2^cd^	70.5 ± 3.2^c^	51.8 ± 6.4^c^	20.5 ± 5.1^cd^	5.3 ± 5.9
*rod1 fae1* (+T, -4)	92.6 ± 5.5^a^	90.9 + 7.5	75.3 ± 4.2^bc^	51.3 ± 6.0^ab^	33.6 ± 5.7^b^	13.3 ± 3.2^ab^	6.8 ± 4.4
*fad2–4 fae1*(+A, -4)	83.2 ± 4.4^b^	93.4 ± 4.4	85.4 ± 5.1^d^	70.8 ± 7.9^c^	57.4 ± 5.0^c^	21.9 ± 3.5^cd^	8.3 ± 4.5
*fad2–5 fae1* (-2, -4)	84.1 ± 4.1^b^	92.9 ± 5.3	84.0 ± 7.7^d^	75.1 ± 9.0^c^	54.5 ± 6.6^c^	22.9 ± 4.0^d^	9.2 ± 6.5
Source of Variation	*F*_7.50_	*F*_7,50_	*F*_7,50_	*F*_7,50_	*F*_7,50_	*F*_7,50_	*F*_7,50_
Type	25.07****	0.50	21.64****	33.94****	68.89****	15.10****	1.85

Data from Experiment 2. Flowering plants were exposed to 7-hour temperature treatments ranging from 20 °C to 34 °C, in wild-type *Thlaspi arvense* (Spring 32-10) and seven gene-edited lines. Temperatures were first set to 20 °C, then progressively raised by 2-3 °C each day, and pollen was collected on consecutive days (at the end of the peak temperature period, at the equivalent of 13 hours after dawn). Values represent mean percents ± standard deviations. Means with the same letter are not statistically significantly different following Tukey’s test. In the analysis of variance, F-values are reported for the effect of genotype (“Type”) at each temperature; **** indicates statistical significance at α = 0.0001. Sample size ranged from *n* = 4 to 8 (average of *n* = 7).

**Table 2:** Thiobarbituric acid reactive substances (TBARS) content in silicles and leaves of non-stressed plants (Control) versus heat-stressed plants (HT).

Line	Treatment	Silicle	Leaf	Silicle	Leaf
		Experiment 1	Experiment 1	Experiment 2	Experiment 2
		nmol g^-1^			
Spring 32 (WT)	Control	116.0 ± 11.3^a^	137.8 ± 19.9^a^	216.5 ± 41.4^c^	245.4 ± 53.7^a-d^
*rod1* (+T)	Control	122.0 ± 33.9^a^	138. 2 ± 15.6^a^	205.6 ± 48.4^bc^	258.1 ± 47.2^b-d^
*fae1* (-4)	Control	119.3 ± 33.5^a^	155.8 ± 5.4^a^	227.3 ± 49.4^c^	210.4 ± 44.6^a-d^
*fad2- 4* (+A)	Control	201.0 ± 37.2^b^	232.9 ± 24.5^b^	255.1 ± 82.3^cd^	220.0 ± 20.8^a-d^
*fad2- 6* (-29)	Control	211.0± 33.8^b^	224.1 ± 29.2^b^	235.0 ± 40.3^c^	273.8 ± 48.0^cd^
*rod1 fae1* (+T, -4)	Control	115.8 ± 33.9^a^	156.0 ± 19.3^a^	189.8 ± 53.2^a-c^	239.4 ± 54.9^a-d^
*fad2–4 fae1* (+A, -4)	Control	221.9 ± 23.7^b^	245.5 ± 28.3^b^	237.1 ± 22.6^cd^	286.7 ± 85.0^de^
*fad2–5 fae1* (-2, -4)	Control			210.9 ± 43.7^c^	239.5 + 71.0^a-d^
Spring 32 (WT)	HT	310.9 ± 21.1^d^	370.0 ± 48.2^c^	379.0 ± 46.0^e^	395.5 ± 25.8^f^
*rod1* (+T)	HT	234.5 ± 11.8^bc^	366.4 ± 33.7^c^	327.5 ± 53.4^de^	372.0 ± 51.2^ef^
*fae1* (-4)	HT	301.3 ± 32.7^cd^	408.3 ± 64.7^c^	409.6 ± 35.7^e^	369.1 ± 54.5^ef^
*fad2- 4* (+A)	HT	106.8 ± 29.5^a^	172.6 ± 29.2^ab^	137.1 + 21.8^ab^	178.7 ± 21.4^ab^
*fad2* (-29)	HT	106.7 ± 16.8^a^	153.1 ± 31.0^a^	132.9 ± 37.3^a^	161.2 ± 22.2^a^
*rod1 fae1* (+T, -4)	HT	300.7 ± 65.4^cd^	394.8 ± 61.0^c^	361.1 ± 37.0^e^	407.8 ± 41.6^f^
*fad2–4 fae1* (+A, -4)	HT	102.5 ± 13.9^a^	150.5 ± 20.8^a^	144.2 ± 16.4^ab^	203.6 ± 39.4^a-c^
*fad2–5 fae1* (-2, -4)	HT			131.6 ± 27.6^a^	191.4 ± 33.9^ab^
Source of Variation		*F*_13, 89_	*F*_13, 93_	*F*_15, 98_	*F*_15, 96_
Mutation		7.84****	14.37****	23.79****	18.34****
Temperature		44.46****	205.17****	2.70	16.39***
Mutation x Temperature		90.42****	99.26****	33.62****	21.44****

Numbers represent means ± standard deviations. Data are from two sequential growth chamber experiments. HT plants were grown until flowering at 20 °C. Temperatures were then progressively raised by 2 – 3 °C per day up to a 34 °C daytime high, held at 34 °C for 9 days, then reduced by 2 – 3 °C per day back to 20 °C. Control plants were grown at 20 °C throughout. Sample size varied from *n* = 6 to *n* = 15 with an average of *n* = 7 per treatment x mutation combination in Experiment 1, and from *n*  = 6 to *n* = 9 with an average of *n* = 7 in Experiment 2. Samples were collected on day 9 of the 34 °C treatment, immediately before the ramp-down in temperature. Means with the same letter are not statistically significantly different following Tukey’s test. Symbols ‘***’ and ‘****’ represent statistical significance at α= 0.001 and 0.0001 respectively.

By contrast, *fad2* knockout did not protect pollen viability at very high temperature (34 °C; *t* = 0.58, *p* > 0.99 for the *fad 2–6* vs. wild type comparison) and had negative effects at “control” temperature (20 °C), compared to wild type. At 20 °C, in Experiment 2, viability was 96 - 100% in wild-type plants and only 80 - 85% in *fad2–6* plants (*t* = 8.01, *p* < 0.0001). Pollen viability for the *fad2* knockout plants was highest at 23 °C. *rod1* knockout also had no protective effect on pollen viability at very high temperature (*t* = 1.08, *p* = 0.96; 34 °C; **Table 1**).

In general, *fae1* knockout did not affect pollen viability in either experiment. *fae1* mutant plants performed similarly to wild type, while *fad2 fae1* mutant plants performed similarly to *fad2* mutant plants (**Table 1**). No differences, at any temperature or in either experiment, were seen between the two *fad2* knockout mutations (either alone or in combination with *fae1*).

### TBARS content following high temperature treatment

In both Experiment 1 and Experiment 2, TBARS content was tested in leaves (containing mostly membrane lipids) and whole silicles containing seeds (comprising both membrane lipids and TAG). The TBARS content was strongly affected by the interaction of mutation and temperature, indicating that the higher-oleic, decreased PUFA lines responded more favorably to HT (**Table 2**). Across both experiments, *fad2* and *fad2 fae1* lines had much lower TBARS content than wild type following HT (34 °C), in both leaf and silicle tissue. By contrast, under control conditions (20 °C throughout), the *fad2* and *fad2 fae1* lines had either higher TBARS than wild type (in Experiment 1) or similar TBARS levels (in Experiment 2).

In Experiment 1, for example, silicle TBARS content was 293–329 nmol g^-1^ fresh weight in the wild type at HT, but was 66% lower in *fad2* lines (e.g. 95–119 nmol g^-1^ in the *fad2–6* line; *t* = 13.40, *p* < 0.0001) and 67% lower in the *fad2 fae1* line (88–117 nmol g^-1^; *t* = 12.63, *p* < 0.0001). By contrast, in the control treatment, silicle TBARS content was 82% higher than wild type in the *fad2–6* line (193–229 nmol g^-1^; *t* = 7.32, *p* < 0.0001) and 91% higher in *fad2 fae1* plants (197–247 nmol g^-1^; *t* = 6.85, *p* < 0.0001). Leaf TBARS levels showed similar effects although of smaller magnitude. At HT, leaf TBARS was 325–415 nmol g^-1^ in wild type and was 59% lower in *fad2–6* plants (129–177 nmol g^-1^; *t* = 12.71, *p* < 0.0001) and in *fad2 fae1* plants (133–168 nmol g^-1^; *t* = 12.50, *p* < 0.0001). By contrast, they were 63% higher (208–240 nmol g^-1^; *t* = 6.92, *p* < 0.0001) and 78% higher (219–272 nmol g^-1^; *t* = 7.16, *p* < 0.0001) than wild type (105–126 nmol g^-1^), respectively, under control (20 °C) conditions.

Silicles of *rod1* plants also showed lower TBARS buildup at HT, but effects were smaller and less consistent than in *fad2* plants. Specifically, at HT, *rod1* plants had 25% lower silicle TBARS than wild type in Experiment 1 (222–247 nmol g^-1^; *t* = 3.49, *p* = 0.044) but performed similarly to wild type in Experiment 2: in both experiments, TBARS levels were equivalent to wild type under control conditions. No differences between *rod1* and wild type leaf tissues were seen in either experiment. No differences were evident between *fae1* and wild type, between *rod1 fae1* and *rod1* or between *fad2 fae1* and *fad2* plants.

### Proline accumulation following high temperature treatment

All lines, in both experiments, saw increases in proline content at HT, in both leaves and silicles (**Table 3**). However, these increases were much smaller in the *fad2* and *fad2 fae1* lines. For example, in Experiment 1, wild type plants saw silicle proline levels increase 7.9-fold at HT, from 236–396 to 2212–2792 nmol g^-1^ fresh weight (*t* = 23.03, *p* < 0.0001) and leaf proline increase 6.1-fold from 418–639 to 2943–3477 nmol g^-1^ (*t* = 18.87, *p* < 0.0001) By contrast, in the *fad2–6* line, proline increased only 3.0-fold in silicles (from 236–461 to 975–1157 nmol g^-1^; *t* = 10.83, *p* < 0.0001), and 3.3-fold in leaves (from 325–420 to 1029–1428 nmol g^-1^; *t* = 9.60, *p* < 0.0001), respectively. Likewise, in the *fad2 fae1* line, silicle proline increased only 3.1-fold at HT (from 256–432 to 955–1146 nmol g^-1^; *t* = 8.56, *p* < 0.0001) while leaf proline increased only 3.2-fold (from 325–478 to 1024–1550 nmol g^-1^; *t* = 7.96, *p* < 0.0001). Alternatively, under HT, *fad2–6* plants had 57% lower silicle proline (*t* = 12.32, *p* < 0.0001) and 62% lower leaf proline compared to wild type (*t* = 12.77, *p* < 0.0001), while under control conditions these did not differ from wild type. Similar trends were evident for the *fad2 fae1* plants. Leaf and silicle proline content in *rod1, fae1*, and *rod1 fae1* plants did not differ from wild type under either HT or control conditions and showed similar trends to wild type. Wild type plants had 67% more proline in leaf tissue than in silicles under normal “control” temperature (*t* = 3.57, *df* = 15, *p* = 0.003) and 29% more under HT (*t* = 4.23, *df* = 15, *p* = 0.0007).

**Table 3:** Proline content (nmol g^-1^) in silicles and leaves of non-stressed plants (Control) versus heat-stressed plants (HT).

Line	Stress	Silicles	Leaves	Silicles	Leaves
		Experiment 1	Experiment 1	Experiment 2	Experiment 2
		nmol g^-1^			
Spring 32 (WT)	Control	316 ± 112^a^	528 ± 144^a^	352 ± 100^a^	593 ± 97^a^
*rod1* (+T)	Control	443 ± 105^a^	508 ± 235^a^	437 ± 174^ab^	553 ± 93^a^
*fae1* (-4)	Control	369 ± 107^a^	632 ± 165^a^	344 ± 166^a^	610 ± 153^a^
*fad2- 4* (+A)	Control	398 ± 132^a^	537 ± 169^a^	511 ± 83^ab^	510 ± 91^a^
*fad2- 6* (-29)	Control	353 ± 168^a^	372 ± 71^a^	515 ± 123^ab^	492 ± 64^a^
*rod1 fae1* (+T, -4)	Control	345 ± 64^a^	530 ± 169^a^	348 ± 58^ab^	479 ± 103^a^
*fad2–4 fae1* (+A, -4)	Control	344 ± 95^a^	401 ± 83^a^	570 ± 155^b^	504 ± 46^a^
*fad2–5 fae1* (-2, -4)	Control			507 ± 98^ab^	522 ± 111^a^
Spring 32 (WT)	HT	2502 ± 377^c^	3216 ± 338^c^	2039 ± 253^d^	3616 ± 216^c^
*rod1* (+T)	HT	2633 ± 426^c^	2953 ± 447^c^	1857 ± 283^d^	3260 ± 575^c^
*fae1* (-4)	HT	2660 ± 356^c^	2910 ± 592^c^	2113 ± 289^d^	3411 ± 529^c^
*fad2- 4* (+A)	HT	1060 ± 164^b^	1276 ± 247^b^	970 ± 91^c^	1101 ± 104^b^
*fad2- 6* (-29)	HT	1066 ± 126^b^	1228 ± 297^b^	961 ± 57^c^	1084 ± 214^b^
*rod1 fae1* (+T, -4)	HT	2453 ± 393^c^	2801 ± 533^c^	2128 ± 282^d^	3213 ± 419^c^
*fad2–4 fae1* (+A, -4)	HT	1051 ± 103^b^	1287 ± 314^b^	935 ± 60^c^	1113 + 252^b^
*fad2–5 fae1* (-2, -4)	HT			937 ± 71^c^	1046 ± 213^b^
Source of Variation		*F*_13, 96_	*F*_13, 99_	*F*_15, 101_	*F*_15, 104_
Mutation		37.13****	39.40****	10.66****	89.04****
Temperature		1278.98****	1103.24****	608.78****	1770.44****
Mutation x Temperature		42.58****	23.40****	24.45****	73.89****

Numbers represent means ± standard deviations. Data are from two sequential growth chamber experiments. HT plants were grown until flowering at 20 °C; temperatures were then progressively raised by 2 – 3 °C per day up to a 34 °C daytime high, held at 34 °C for 9 days, then reduced by 2 – 3 °C per day back to 20 °C. Control plants were grown at 20 °C throughout. Sample size varied from *n* = 6 to *n* = 12 with an average of *n* = 8 per treatment x mutation combination in Experiment 1, and from *n*  = 6 to *n* = 9 with an average of *n* = 7 in Experiment 2. Samples for proline content were collected on day 9 of the 34 °C treatment, immediately before the ramp-down in temperature. Means with the same letter are not statistically significantly different following Tukey’s test. Symbol ‘****’ represents statistical significance at α= 0.0001.

The same overall trends were evident in Experiment 2. As in the previous experiment, all lines experienced increased proline levels at HT, but the increases were much smaller in *fad2* and *fad2 fae1* plants than in wild type or the other mutant lines. No differences were seen between the two *fad2* mutant lines in either experiment, or between the two *fad2 fae1* lines in Experiment 2.

### Peroxide content following high temperature treatment

Peroxide content was measured as an indicator of reactive oxygen species (ROS) buildup. Under high temperature, peroxide levels increased in leaves and silicles of wild-type plants, while decreasing in the *fad2* or *fad2 fae1* plants (**Table 4**). The *rod1, fae1* and *rod1 fae1* plants generally showed similar trends to wild type. These trends were consistent across both experiments. In Experiment 1, for example, wild type plants had a 5.1-fold increase in silicle peroxide levels (*t* = 23. 14, *p* < 0.0001) and a 5.7-fold increase in leaf peroxide content (*t* = 21.95, *p* < 0.0001) in the high-temperature treatment, while *fad2–6* and *fad2 fae1* plants had on average 44% decreases in silicle peroxide content (*t* = 8.03, *p* < 0.0001) and in leaf peroxide content (*t* = 7.38, *p* < 0.0001) respectively.

**Table 4:** Hydrogen peroxide content (nmol g^-1^) in silicles and leaves of non-stressed plants (Control) versus heat-stressed plants (HT).

Line	Treatment	Silicle	Leaf	Silicle	Leaf
		Experiment 1	Experiment 1	Experiment 2	Experiment 2
		nmol g^-1^			
Spring 32 (WT)	Control	10.2 ± 1.4^a^	10.9 ± 1.3^ab^	10.6 ± 2.2^ab^	11.7 ± 1.5^b^
*rod1* (+T)	Control	11.2 ± 1.9^a^	9.8 ± 1.4^a^	10.7 ± 2.2^ab^	12.2 ± 0.9^b^
*fae1* (-4)	Control	9.6 ± 1.6^a^	11.7 ± 2.0^ab^	10.1 ± 1.7^ab^	12.3 ± 1.9^b^
*fad2- 4* (+A)	Control	20.9 ± 2.3^b^	25.1 ± 4.2^c^	20.1 ± 4.5^d^	19.9 ± 2.7^c^
*fad2- 6* (-29)	Control	19.5 ± 2.9^b^	23.4 ± 2.5^c^	21.7 ± 6.9^d^	19.3 ± 2.7^c^
*rod1 fae1* (+T, -4)	Control	10.8 ± 2.1^a^	10.9 ± 2.2^ab^	9.5 ± 2.4^ab^	12.1 ± 3.9^b^
*fad2–4 fae1* (+A, -4)	Control	22.5 ± 1.8^b^	26.4 ± 3.2^c^	23.9 ± 6.0^d^	23.8 ± 6.5^c^
*fad2–5 fae1* (-2, -4)	Control			16.5 ± 2.7^cd^	18.5 ± 1.7^c^
Spring 32 (WT)	HT	52.2 ± 5.4^c^	61.9 ± 11.2^d^	55.3 ± 6.9^e^	46.3 ± 5.2^d^
*rod1* (+T)	HT	49.3 ± 3.8^c^	54.9 ± 11.8^d^	50.7 ± 8.5^e^	36.6 ± 2.2^d^
*fae1* (-4)	HT	52.1 ± 4.9^c^	51.5 ± 6.3^d^	52.3 ± 9.1^e^	42.0 ± 6.7^d^
*fad2- 4* (+A)	HT	10.3 ± 1.4^a^	13.3 ± 1.8^b^	11.8 + 3.1^a-c^	12.0 ± 2.5^b^
*fad2- 6* (-29)	HT	11.1 ± 1.5^a^	13.1 ± 1.2^b^	7.9 ± 1.5^a^	9.4 ± 1.9^ab^
*rod1 fae1* (+T, -4)	HT	52.9 ± 5.3^c^	58.2 ± 7.8^d^	56.5 ± 12.8^e^	38.4 ± 1.4^d^
*fad2–4 fae1* (+A, -4)	HT	11.0 ± 1.9^a^	11.8 ± 2.4^ab^	12.9 ± 3.0^cb^	8.3 ± 3.1^a^
*fad2–5 fae1* (-2, -4)	HT			10.8 ± 2.3^ab^	10.7 ± 1.5^ab^
Source of Variation		*F*_13, 92_	*F*_13, 92_	*F*_15, 102_	*F*_15, 102_
Mutation		42.21****	19.64****	22.93****	26.57****
Temperature		525.98****	454.67***	164.55****	55.29****
Mutation x Temperature		286.15****	241.25****	122.74****	121.03****

Numbers represent means ± standard deviations. Data are from two sequential growth chamber experiments. HT plants were grown until flowering at 20 °C. Temperatures were then progressively raised by 2 – 3 °C per day up to a 34 °C daytime high, held at 34 °C for 9 days, then reduced by 2 – 3 °C per day back to 20 °C. Control plants were grown at 20 °C throughout. Sample size varied from *n* = 6 to *n* = 8 in both experiments. Samples for MDA content were collected on day 9 of the 34 °C treatment, immediately before the ramp-down in temperature. Means with the same letter are not statistically significantly different following Tukey’s test. Symbols ‘***’ and ‘****’ represent statistical significance at α= 0.001 and 0.0001 respectively.

### Effects of high temperature treatment on seed yield

**Figure 6:**
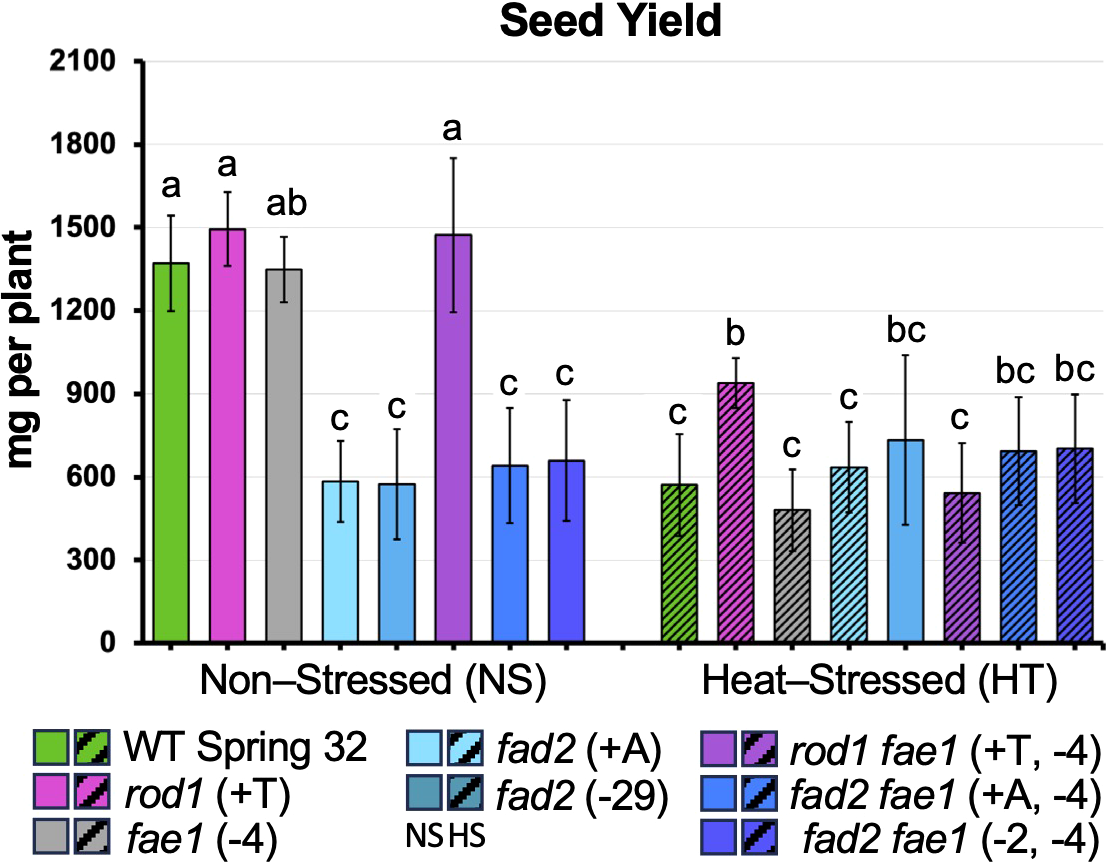
Seed yield of plants that were non-stressed (NS; solid columns) or heat-stressed (HT; hatched columns). Data from Experiment 2. Heat-stressed plants were grown until flowering at 20 °C. Temperatures were then progressively raised by 2 – 3 °C per day up to a 34 °C daytime high, held at 34°C for 9 days, then reduced by 2 – 3 °C per day back to 20 °C. Non-stressed plants were grown at 20 °C throughout. Bars represent standard deviations. Means with the same letter are not statistically significantly different, following Tukey’s HSD test. Sample size varied from n = 8 to n = 22 with an average of n = 12.

Consistent with the data on pollen viability, the *fad2* and *rod1* mutations ameliorated the negative effects of HT on seed yield (**Figure 6**; **Supplementary Figure 4**). We present the data for both experiments separately, as stated above. Experiment 2 is discussed in more detail as it had more lines, but trends were similar across both experiments. In Experiment 2, seed yield varied among the lines (*F*_7,170_ = 23.66, *p* < 0.0001), and was affected by temperature (*F*_1,170_ = 121.98, *p* < 0.0001). There was a strong interaction between mutation and temperature (*F*_7,170_ = 28.92, *p* < 0.0001; **Figure 6**). Wild-type plants saw a 58% decline in seed yield under HT, from 1284–1456 to 473–670 mg plant^-1^ (*t* = 11.12, *p* < 0.0001), and similar declines were seen for *fae1* plants (64% decrease, from 1250–1448 to 357–604 mg plant^-1^: *t* = 8.22, *p* < 0.0001), and *rod1 fae1* plants (63% decrease, from 1274–1671 to 408–677 mg plant^-1^: *t* = 9.47, *p* < 0.0001). By contrast, *rod1* plants had only a 38% decrease in seed yield (from 1399–1590 to 875–1003 mg plant^-1^: *t* = 5.15, *p* < 0.0001) while *fad2* and *fad2 fae1* plants had no decline following HT (e.g. *p* = 0.83 for *fad 2–6* and *p* > 0.99 for both of the *fad2 fae1* lines). The *fad2* and *fad2 fae1* lines had lower seed yield than wild type under control conditions, indicating a yield penalty associated with *fad2* loss of function. However, this yield penalty was fully eliminated under heat stress. For example, *fad 2–6* plants had poorer yield than wild type under nonstressed conditions (446–700 mg plant^-1^; *t* = 10.22, *p* < 0.0001) but performed equivalent to wild type under HT (565–902 mg plant^-1^; *p* = 0.71). Likewise, the *fad 2–5 fae1* line had lower yield than wild type in the control treatment (519–798 mg plant^-1^; *t* = 9.14, *p* < 0.0001), but equivalent to wild type following HT (578–827 mg plant^-1^: *p* = 0.96). The two *fad2* lines did not differ from each other, nor did the two *fad2 fae1* lines, either under control or HT conditions (*p* > 0.99 for all comparisons).

Similar trends were seen in Experiment 1 (**Supplementary Figure 4****).** Seed yield was affected by line (*F*_6,138_ = 29.36, *p* < 0.0001), by temperature (*F*_1,138_ = 141.91, *p* < 0.0001), and by the interaction of the two (*F*_6,138_ = 29.17, *p* = 0.0001). As before, *fad2* and *fad2 fae1* plants suffered no decrease in yield following HT while other lines did, and the yield penalty in *fad2* and *fad2 fae1* plants, relative to wild type, was present under control conditions but was fully eliminated following HT. In Experiments 3 and 4, seed yield and total dry biomass were quantified under HT as well as control conditions. As described above, for space reasons, Experiment 4 was done in two batches: 4a included wild type, *fad2* and *fad2 fae1* while 4b included wild type, *rod1, fae1* and *rod1 fae1*. Across both experiments, HT imposed at the beginning of flowering caused large reductions in both seed yield and biomass for wild type, as well as for *fae1*, *rod1* and *fae1* plants, but not for *fad2* or *fad2 fae1* plants (**Table 5**). Results were generally consistent across the two experiments. In Experiment 4a, for example, HT caused wild type plants to experience a 41% decrease in biomass (from 2693–3297 to 1551–1818 mg plant^-1^; *t* = 8.23, p < 0.0001) and a 59% decrease in yield (from 968–1132 to 379–457 mg plant^-1^; *t* = 13.12, *p* < 0.0001). In contrast, the *fad2* and *fad2 fae1* lines saw no decrease in either yield or biomass under HT. For example, for *fad2 - 6*, yield was 471–554 mg plant^-1^ under control conditions and 424–563 mg plant^-1^ following HT (*p* > 0.99); biomass was 1765–2066 mg plant^-1^ under control conditions and 1904–2315 mg plant^-1^ following HT (*p* > 0.95). For *fad2–5 fae1*, yield was 511–669 mg plant^-1^ under control conditions and 495–628 mg plant^-1^ following HT (*p* > 0.99); biomass was 1736–2173 mg plant^-1^ under control conditions and 2104–2567 mg plant^-1^ following HT (*p* = 0.24). Alternatively stated, *fad2–6* plants had only 49% of wild type seed yield and 64% of wild type biomass at normal temperature but performed equivalent to wild type following HT. Similar results were seen in Experiment 3.

**Table 5:** Senesced biomass and seed yields of non-stressed plants (Control) versus heat-stressed plants (HT).

Line	Stress	Biomass	Seed	Biomass	Seed	Biomass	Seed
		Exp 3	Exp 3	Exp 4a	Exp 4a	Exp 4b	Exp 4b
		mg plant^−1^					
Spring 32 (WT)	Control	3113 ± 182^a^	1127 ± 157^a^	2995 ± 942^a^	1050 ± 255^a^	3071 ± 835^a^	984 ± 284^a^
*rod1* (+T)	Control	3038 ± 289^a^	1092 ± 131^a^			2974 ± 769^a^	1003 ± 298^a^
*fae1* (-4)	Control	3004 ± 172^a^	1062 ± 155^a^			3021 ± 1086^a^	1004 ± 318^a^
*fad2- 4* (+A)	Control	1902 ± 140^b^	477 ± 64^b^	1935 ± 594^bc^	568 ± 179^b^		
*fad2- 6* (-29)	Control	1932 ± 155^b^	452 ± 62^b^	1916 ± 475^bc^	513 ± 132^b^		
*rod1 fae1* (+T, -4)	Control	3035 ± 229^a^	1050 ± 179^a^			2906 ± 991^a^	1046 ± 391^a^
*fad2–4 fae1* (+A, -4)	Control	2039 ± 145^b^	423 ± 127^b^	1883 ± 422^bc^	522 ± 144^bc^		
*fad2–5 fae1* (-2, -4)	Control	1967 + 188^b^	458 + 80^b^	1954 ± 672^bc^	590 ± 242^b^		
Spring 32 (WT)	HT	1957 ± 154^b^	424 ± 112^b^	1685 ± 417^c^	418 ± 121^c^	1999 ± 635^b^	401 ± 137^b^
*rod1* (+T)	HT	2027 ± 122^b^	468 ± 168^b^			2119 ± 888^b^	436 ± 217^b^
*fae1* (-4)	HT	2022 ± 260^b^	400 ± 153^b^			2171 ± 803^b^	418 ± 122^b^
*fad2- 4* (+A)	HT	2066 ± 168^b^	432 ± 41^b^	2058 ± 880^bc^	518 ± 264^bc^		
*fad2- 6* (-29)	HT	2005 ± 164^b^	478 ± 58^b^	2110 ± 650^bc^	493 ± 220^bc^	2016 ± 450^b^	424 ± 155^b^
*rod1 fae1* (+T, -4)	HT	1906 ± 138^b^	393 ± 78^b^				
*fad2–4 fae1* (+A, -4)	HT	2084 ± 130^b^	433 ± 73^b^	2055 ± 750^bc^	478 ± 193^bc^		
*fad2–5 fae1* (-2, -4)	HT	2073 ± 162^b^	456 ± 58^b^	2336 ± 736^b^	562 ± 211^b^		
Source of Variation		*F*_15, 176_	*F*_15, 176_	*F*_9, 388_	*F*_9, 384_	*F*_7, 296_	*F*_7, 296_
Mutation		47.18****	37.81****	3.39**	14.21****	0.23	0.23
Temperature		298.56****	352.61****	1.78	60.60****	90.71****	423.64****
Mutation x Temperature		65.03****	50.38****	18.861****	29.37****	0.35	0.03

Numbers represent means ± standard deviations. Data are from two sequential growth chamber experiments. Experiment 4, for space reasons, was conducted as two separate batches (4a and 4b) with wild type controls in both. HT plants were grown until just before flowering at 20 °C. temperatures were then raised to 28 °C / 22 °C (day/night) for 1 day, raised to 34 °C / 28 °C (day/night) and held for 14 days, then reduced back to 28 °C / 22 °C for 1 day and then returned to the 20 °C chamber for the rest of their life cycle. Control plants were grown at 20 °C throughout. Sample size was *n*  = 12 in Experiment 3, varied from *n* = 37 to *n* = 40 with an average of *n* = 39 per treatment x mutation combination in Experiment 4a, and varied from *n*  = 32 to *n* = 40 with an average of *n* = 37 in Experiment 4b. Means with the same letter are not statistically significantly different following Tukey’s test. Symbols ‘**’ and ‘****’ represent statistical significance at α = 0.05, 0.01, 0.001 and 0.0001 respectively.

In contrast to Experiments 1 and 2, the *rod1* mutation did not confer an advantage at HT in terms of yield or biomass, with *rod1* having similar yield and biomass to wild type in both experiments (**Table 5**). Reproductive allocation (seed yield as a fraction of dry biomass) was negatively affected by heat stress and was also lower, under control conditions, in *fad2* and *fad2 fae1* lines than in wild type or the *fae1*, *rod1* and *rod1 fae1* lines. In Experiment 4a, for example, reproductive allocation in wild type decreased from 34 - 39% at normal temperature to 24 - 26% following HT (*t* = 10.47, *p* < 0.0001), while in *fad2–6* it was only 26 - 28% under normal temperature (*t* = 8.37, *p* < 0.0001 for the *fad2–6* vs. wild type comparison). At HT, these effects of lower reproductive allocation and improved heat tolerance counteracted each other (i.e. reproductive allocation in *fad2* mutant lines decreased less than in wild type), so that the *fad2* and *fad2 fae1* lines had similar reproductive allocation to wild type.

### Differential effects on male vs. female fertility

Both male and female fertility were strongly negatively affected by transient HT (six hours at 34 °C), with even sharper reductions in female fertility compared to male (**Table 6**). In crosses where the female (pollen-recipient) flowers experienced HT only 0 - 14% of crosses were successful, compared to a 9 - 42% success rate when the male (pollen-donor) flowers were heat stressed (*t* = 2.47, *p* = 0.033, *df* = 10 for the comparison of male-stressed vs. female-stressed: *n* = 8 in all cases). Interestingly, the fraction of successful crosses where the pollen donor experienced HT (9 - 42%) was much higher than the fraction of viable pollen (1 - 12%, from **Table 1**; **Supplementary Table 1**). This could indicate either that cumulative, long-term increases in temperature cause more damage to pollen than short bursts of very high temperature, or else that even a small fraction of viable pollen is enough to ensure that ca. 25% of flowers can be successfully fertilized.

**Table 6:** Cross-pollination success rates when crossing pollen from a heat-stressed plant with a non-stressed pistil and vice versa.

Treatment x sex	Successful crosses per plant (%)	
	Non-stressed (male)	Heat-stressed (male)
Control (Female)	82.6 ± 18.1	25.5 ± 20.0
HT (Female)	5.7 ± 10.9	---

Treatment x sex	Successful crosses across experiment (%)	
	Non-stressed (male)	Heat-stressed (male)
Control (Female)	82.9 ± 37.6	32.4 ± 46.7
HT (Female)	8.1 ± 27.3	---

Mean percentage (with standard deviation) of crosses between separate wild-type pennycress (Spring 32-10) plants that produced seed, when either the male parent, the female parent, or neither parent, was subjected to a short-term, 6-hour high temperature (HT) treatment. The HT treatment (34 °C) was imposed between 10 a.m. and 4 p.m. Recipient flowers were emasculated immediately before heat treatment and crosses were performed immediately afterwards. Control plants were kept at 21 °C throughout. Eight plants were included in each treatment, with 3–7 crosses attempted per plant. The top two rows (“crosses per plant”) treat every plant as a single replicate and estimate the typical percentage of successful crosses per plant. The lower two rows (“crosses across experiment”) pool all the crosses across the eight plants and estimate the percentage of successful crosses across the experiment, using the binomial standard deviation.

### Effects of high temperature on seed viability

Seed viability (expressed as the ability of seeds to germinate after 14 days on agar plates) was unaffected either by HT (*p* = 0.36), by the high-oleic/reduced PUFAs mutations (*p* = 0.39), or by the interaction of the two (*p* = 0.51). Germination rates at day 14 were 88 - 93% across all treatment combinations. For wild-type plants, no difference in germination rate was seen on any day of the time course.

## Discussion

At high temperatures (28 °C to 34 °C; HT), the pennycress *fad2* and *rod1* lines suffered less lipid peroxidation in silicles than wild type, maintained greater pollen viability, and experienced less of a decline in seed yield. The benefits of these mutations are likely due to the reduced PUFA content in membrane lipids which produces more rigid membranes better suited to higher temperatures and less substrate for lipid peroxidation. These effects were greater in *fad2* than in *rod1*. In addition, the *fad2* plants also showed less buildup of leaf TBARS, silicle and leaf peroxide content, and silicle and leaf proline content (compared to wild type) under heat stress, whereas *rod1* did not. The fact that effects were stronger and seen across more parameters in *fad2* than in *rod1* plants likely reflects the differential role of each enzyme on membrane lipid and TAG fatty acid compositions. FAD2 is involved in total ER localized fatty acid desaturation, but ROD1 is mostly involved in transfer of PUFA from PC to the DAG substrate for TAG synthesis (Li-Beisson et al., 2013). Thus, *fad2* affects fatty acid composition in membranes and TAG in all tissues, whereas *rod1* mostly affects TAG fatty acid composition in the tissues that accumulate TAG, which are predominantly seed embryos and pollen. Since pollen TAG turnover is used to make pollen tube membrane lipids (Ischebeck, 2016) and pollen in both the *fad2* and *rod1* mutant backgrounds contain less PUFA (**Figure 4**), the pollen tube membrane lipids likely have less PUFA than WT. These results suggest that while pennycress suffers sharp declines in pollen viability at even moderately elevated temperatures, and large yield declines following extreme high temperature (34 °C), these reproductive losses can be eliminated by *fad2* knockout and lessened by *rod1* knockout. The large effects on TBARS content, coupled with the fact that MDA negatively impacts pollen viability (Jia et al., 2017) suggests that decreases in membrane and lipid peroxidation could be one mechanism by which lower PUFAs content ameliorates the negative effects of HT.

To the best of our knowledge, this is the first study to directly investigate the effects of *fad2* and *rod1* knockdown on HT responses during flowering. However, our findings are consistent with previously documented effects of lipid saturation level on membrane stability and lipid peroxidation under heat stress. For example, Yamauchi et al. (2008) investigated *fad3 fad7 fad8* triple mutants of *Arabidopsis*, which lack the ability to produce 18:3 in both chloroplasts and non-photosynthetic tissues. These authors found that MDA levels were lower in the triple mutants under both control and heat stressed conditions, that the triple mutants had undetectable levels of MDA-damaged protein under heat stress (implying greater thermotolerance), and that *in vitro* MDA production and protein damage by MDA were higher at 37 °C compared to 25 °C. Importantly, linolenic acid, *in vitro*, was also found to be a much more effective source of MDA than linoleic or oleic acid (consistently with Kishida et al., 1993). This can help explain why, in our study, large decreases in lipid peroxidation (assayed as TBARS) were seen in *fad2* and *rod1* lines (e.g. 63% and 49% decreases relative to wild type, in HT-exposed silicles in Experiment 2), even though decreases in overall unsaturation were more modest. Our findings are also consistent with recent work exploring effects of fatty acid saturation on heat tolerance in wheat (Yu et al., 2026), soybean (Toyinbo et al., 2025), *Pinellia* (Zhang et al., 2021), and *Arabidopsis* (Larkindale et al., 2005; Wallis and Browse, 2002). The fact that *fad2* plans also experienced lower hydrogen peroxide (H_2_O_2_) levels under heat stress suggests that other mechanisms may additionally contribute to the reductions in lipid peroxidation. Hydrogen peroxide (H_2_O_2_) levels reflect reactive oxygen species (ROS) buildup upstream of the lipid peroxidation reaction, and reduced H_2_O_2_ levels in the low-PUFAs lines could reflect a better ability to maintain optimal membrane rigidity under heat stress. The fact that we observed smaller increases in proline in the more heat tolerant *fad2* and *fad2 fae1* lines is also interesting, since it suggests that the mechanism of improved HT tolerance in these lines is not due to an increased ability to accumulate proline. Proline is produced in response to a wide range of abiotic stresses (Szabados and Savouré, 2010; Raza et al., 2023) and serves a variety of protective functions including as a chemical chaperone stabilizing proteins (Samuel et al., 2000), as a membrane lipid stabilizer, as an osmolyte and as a reactive oxygen species scavenger (Forlani et al., 2019). However, while proline is sometimes viewed as purely beneficial, its direct effects can be complex. Proline can lead to tradeoffs against growth (Maggio et al., 2002), and can sometimes build up to a greater extent, or more quickly, in susceptible than in tolerant cultivars (Bansal and Nagarajan, 1986; Schlafleitner et al., 2007). In *Arabidopsis* seedlings under heat stress, proline overexpression is associated with higher MDA levels and lower survival (Lv et al., 2011). Our results suggest that in this species, proline buildup can be an indicator and symptom of heat stress as well as protection against it (Schlafleitner et al., 2007; Spormann et al., 2023).

Despite the importance of rapid lipid production in pollen viability (Wallis and Browse, 2002; Krawczyk et al., 2022), few studies have specifically examined the effects of 18:2 and 18:3 knockdown on pollen viability, and to the best of our knowledge none have done so under heat stress. McConn and Browse (1996) found that *fad3 fad7 fad8* triple mutants of *Arabidopsis*, with no 18:3 (linolenic acid) content in either leaves or non-photosynthetic tissues, were unable to produce viable pollen. However, male fertility could be restored by applying exogenous linolenic acid, which in part gets converted to jasmonic acid involved in anther development. The authors concluded that as little as 1-2% of total fatty acids in 18:3 form could restore fertility. Our study is consistent with these findings; the *fad2* and *fad2 fae1* pennycress lines had 3-5% 18:2 content in whole flowers and pollen respectively, and 18:3 content of flowers and pollen was well above the critical threshold identified for male fertility in *Arabidopsis* (at about 3%; McConn and Browse, 1996). We did find a negative effect of the pennycress *fad2* mutant on pollen viability at the standard growing temperature of 20 °C. However, as noted, this effect was reversed at moderately high temperatures (26 – 30 °C) where *fad2* plants had a strong advantage.

Beyond pollen viability, this study in pennycress is also consistent with previous studies that explored the effects of desaturases on other aspects of heat tolerance. Wallis and Browse (2002) found improved photosynthetic performance of *fad6* mutants under heat stress, while Murakami et al. (2000) found improved photosynthesis and growth in *Arabidopsis* and tobacco with loss of function of *fad7* and *fad8*. Similarly, knockout of stearic acid desaturase in *Pinellia ternate* led to greater lipid saturation and improved thermotolerance (Zhang et al., 2021). Most recently, in soybean, mutations in *FAD2* and *FAD3* caused improved seed germination under heat stress (Toyinbo et al., 2025), while loss-of-function mutations in the linoleic acid desaturase *FAD8* improved survival and thousand kernel weight under HT stress in wheat (Yu et al., 2026). Conversely, overexpression of *FAD2* was found to improve cold tolerance in poplar (Zhou et al., 2010) and *Arabidopsis* (Wang et al., 2021) while silencing *FAD2* reduced cold tolerance in tea (Pi et al., 2023). In nature, plants commonly increase desaturase expression or PUFAs levels at low temperature (Wei et al., 2006; Xue et al., 2017), or decrease them at high temperature (Narayanan et al., 2020; Zoong Lwe et al., 2020, Zoong Lwe et al., 2021; Rahnama et al., 2024, Rahnama et al., 2026). In some species, more heat tolerant cultivars show a greater ability to decrease 18:3 levels, unsaturation index or *FAD* expression than heat-susceptible varieties, e.g. in peanut (Zoong Lwe et al., 2020) and soybean (Narayanan et al., 2020). It will be interesting to determine if pennycress *fad2* mutants also exhibit a tradeoff between heat and cold tolerance or if pennycress behaves differently perhaps due to its extreme cold tolerance which is not present in the studied species.

An interesting note is that while *fad2* mutant plants had much higher pollen viability than wild type at moderately elevated temperature, they did not have higher seed yield following the HT treatment. Heat stress eliminated the yield penalty (i.e., *fad2* mutants went from 40% of wild-type yield under non-stressed conditions to equivalent to wild type following the HT treatment), but it did not reverse it (i.e. *fad2* plants never had superior yield to wild type). Part of the explanation could be that the large differences in pollen viability between *fad2* and wild-type plants were most important under moderate rather than severe (34 °C) elevated temperature. It is important to note that these plants experienced a 9-day period at 34 °C following the pollen viability tests. During this period, pollen viability would be low in all plants, the *fad2* ones included, and some of the positive effects seen under more moderate HT might be blunted.

While *FAD* has been extensively studied over many years, there have been few studies of the involvement of *ROD1/PDCT* in PUFAs accumulation (Lu et al., 2009; Wickramarathna et al., 2015). To the best of our knowledge, this is one of the first studies to specifically explore the significance of *ROD1* for pollen lipid content and heat stress tolerance, although Mueller et al. (2017) found that *Arabidopsis rod1* seedlings performed similarly to wild type in terms of fatty acid composition under heat stress. Our findings of improved pollen viability and decreased TBARS content in pennycress *rod1* plants under heat stress are particularly promising, because *rod1* knockout mutant plants lack a yield penalty both in growth chamber and field conditions. While the *fad2* knockout mutations in this study had a profound desirable impact on seed oleic acid and PUFAs composition as well as heat tolerance, the yield penalty the mutants suffer make them currently unsuitable for incorporation into commercial pennycress varieties. The differences between the two mutant lines likely are due to *fad2* affecting membrane lipid content in all tissues, whereas *rod1* mostly effects TAG fatty acid content in TAG accumulating tissues, and that pollen TAG can be used to make membranes during pollen tube growth. More modest reductions in seed PUFAs, obtained through *rod1* knockout (Sandgrind et al., 2023) or possibly through partial rather than full loss of function of *fad2* (Park et al., 2021; Neelakandan et al., 2022; Wang et al., 2025) may be commercially viable. One interesting finding, with respect to *rod1*, was that this mutation improved seed yield following HT relative to wild type in Experiments 1–2 but not in Experiments 3 - 4. This may reflect the fact that in Experiments 3 - 4, the severe HT condition (34 °C) was imposed at an earlier stage (throughout the entirety of flowering, beginning at day 27 after transplanting) than in Experiments 1-2 (late flowering and silicle development, beginning at day 34 after transplanting). The effect of *rod1* on mitigating effects of HT may therefore depend on the exact developmental stage at which HT stress occurs.

## Conclusion

Pennycress domestication has made rapid strides over the last decade, and pennycress today stands at the verge of becoming an impactful multi-purpose oilseed-producing intermediate crop. This study builds on previous work to improve quality traits of pennycress seed oil through gene editing for increased oleic acid content. We found that in addition to its effects on seed oil composition, *fad2* loss-of-function also led to large decreases in PUFA content in both floral lipids and pollen lipids. *rod1* loss of function, by contrast, led to decreased PUFA content in pollen lipids while having little effect on whole flowers. The decreases in PUFA levels in pollen and flowers of *fad2* plants led to much greater pollen viability under moderately elevated temperature, as well as to less proline accumulation and less lipid peroxidation following a 9-day high temperature (HT) treatment. *fad2* and *fad2 fae1* plants tolerated HT with no decrease in yield (compared to over 60% yield decrease in wild type), and HT eliminated the yield penalty in *fad2* plants relative to wild type. Smaller improvements in HT tolerance were seen in *rod1* plants, consistent with its intermediate lipid phenotype. In addition to improving pennycress seed oil as both an edible oil and biofuels feedstock, the reductions in membrane lipid and TAG PUFAs content appear to improve heat stress tolerance as an auxiliary benefit.

## Data Availability

The datasets generated and analyzed during the current study are publicly available in the Dryad repository at: https://doi.org/10.5061/dryad.ttdz08mcb.
