# Supplementary material for: Decreases in polyunsaturated fatty acid content improve heat stress tolerance during flowering and silicle development in pennycress (Thlaspi arvense L.)
Source: Front Plant Sci. 2026 May 5;17:1811992. doi: 10.3389/fpls.2026.1811992 (PMC13183578; doi:10.3389/fpls.2026.1811992)
Supplement: Supplementary file 2 [file SupplementaryFile1.pdf]

Supplementary Material

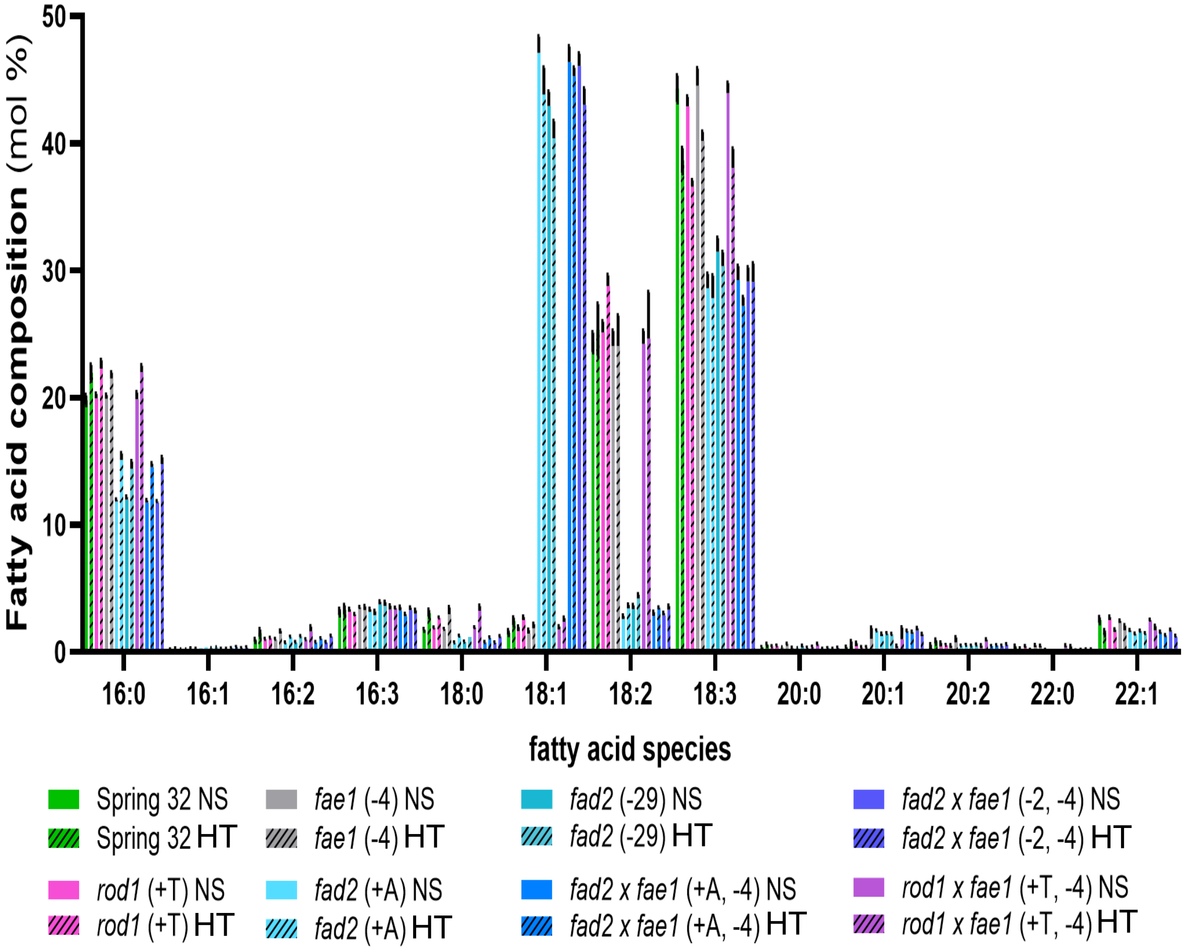

Supplementary Figure 1. Fatty acid composition of entire flowers. Composition of 13 individual fatty acids was compared between heat-stressed (HT) and non-stressed (NS) whole flowers of wild-type (Spring 32-10) and six gene-edited lines with complete loss of function of either the *reduced oleate desaturase 1* (*rod1*), *fatty acid elongase 1* (*fae1*), *fatty acid desaturase 2* (*fad2*), or double homozygous mutant combinations of the *rod1* x *fae1* or *fad2* x *fae1*. Heat-stressed plants were grown until flowering at 20 °C; temperatures were then progressively raised by 2 – 3 °C per day up to a 34 °C daytime high, held at 34 °C for 9 days, then reduced by 2 – 3 °C per day back to 20 °C. Non-stressed plants were grown at 20 °C throughout. Fatty acids are presented as mol % of total FA. Data is presented as mean ± standard deviation with statistical differences measured by ANOVA and the Holm-Šídák multiple comparison test. Means with different letters denote significant differences ($P \leq 0.05$) between lines, sample size n = 5-10. Non-stressed (NS) composition is solid bars, heat-stressed (HT) samples have black hashmarks.

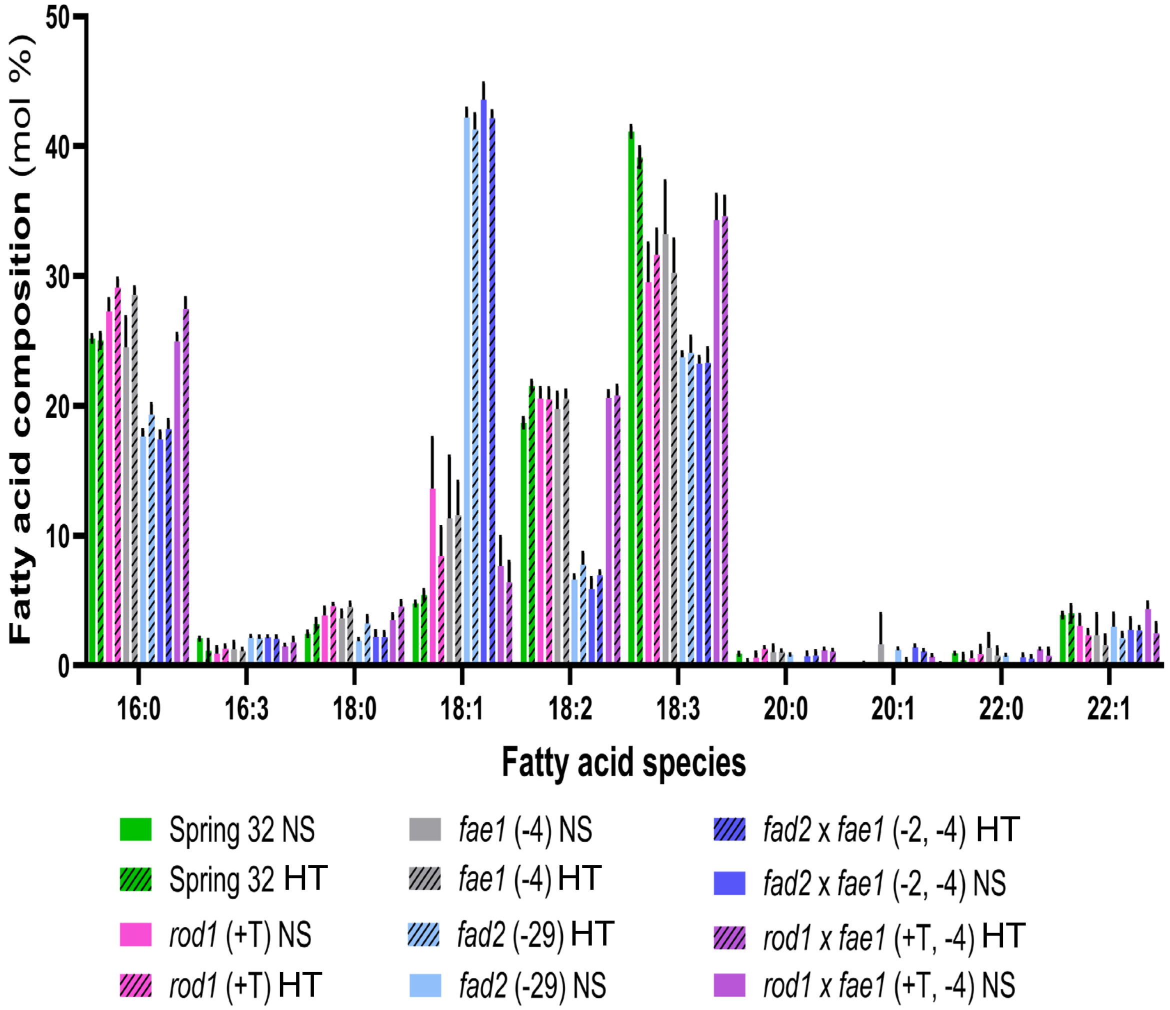

Supplementary Figure 2. Fatty acid composition of pollen enriched fraction. Composition of 13 individual fatty acids was compared between heat-stressed (HT) and non-stressed (NS) pollen-enriched floral extracts of wild-type (Spring 32-10) and six gene-edited lines with loss of function of either the *reduced oleate desaturase 1* (*rod1*), *fatty acid elongase 1* (*fae1*), *fatty acid desaturase 2* (*fad2*), or double mutant combinations of *rod1* x *fae1* or *fad2* x *fae1*. Heat-stressed plants were grown until flowering at 20 °C; temperatures were then progressively raised by 2 – 3 °C per day up to a 34 °C daytime high, held at 34 °C for 9 days, then reduced by 2 – 3 °C per day back to 20 °C. Non-stressed plants were grown at 20 °C throughout. Fatty acids (FA) are presented as mol % of total FA. Data is presented as mean ± standard deviation with statistical differences measured by ANOVA and the Holm-Šídák multiple comparison test. Means with different letters denote significant differences ($P \leq 0.05$) between lines, sample size n = 5-10. Non-stressed (NS) composition is solid bars, heat-stressed (HT) samples have black hashmarks.

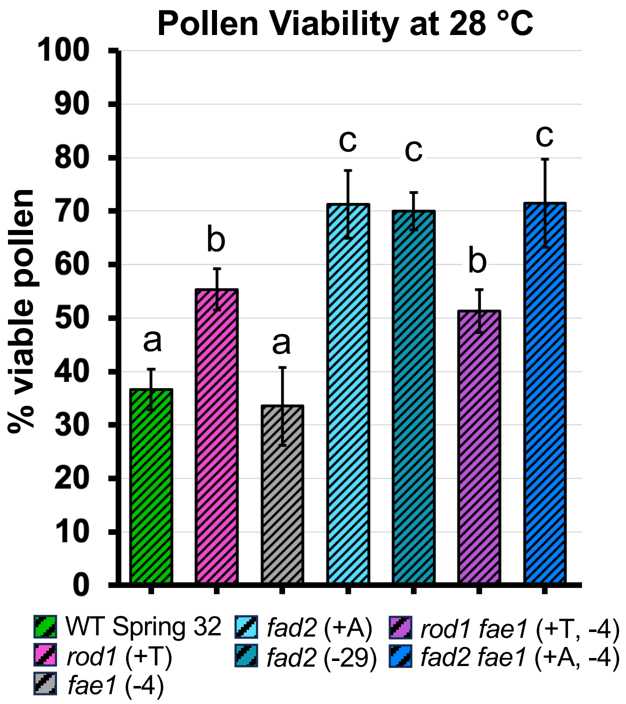

Supplementary Figure 3. Mean pollen viability at elevated temperatures. The data is from Experiment 1, chamber-grown wild-type Spring 32-10 pennycress and six gene-edited lines with loss of function of either the *rod1*, *fae1*, *fad2*, or combinations of *rod1* x *fae1* or *fad2* x *fae1*. The experiment involved progressively increasing temperatures (temperature increases of 2 – 3 °C in daytime highs), on the days when peak daytime temperatures were set at 28 °C. Bars represent standard deviations. Statistical differences were measured by ANOVA and the Tukey multiple comparison test. Means with different letters denote significant differences ($P \leq 0.05$) between lines, sample size n = 4 – 11 with an average of n = 7. See Supplementary Table 1 for pollen viability percentages at other temperatures.

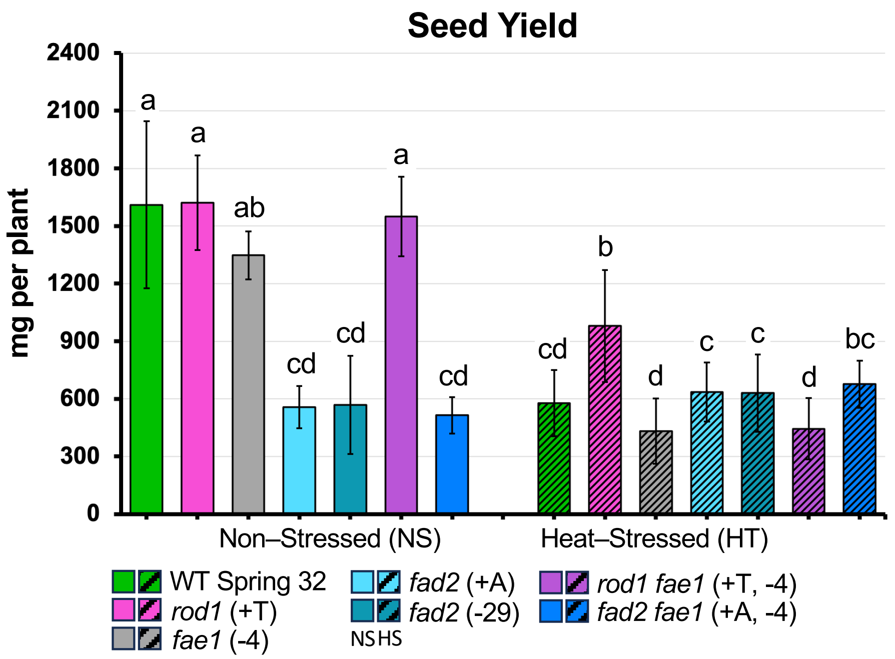

Supplementary Figure 4. Seed yield of plants that were non-stressed (NS; solid columns) or heat-stressed (HT; hatched columns). Data from Experiment 1. Heat-stressed plants were grown until flowering at 20 °C. Temperatures were then progressively raised by 2 – 3 °C per day up to a 34 °C daytime high, held at 34 °C for 9 days, then reduced by 2 – 3 °C per day back to 20 °C. Non-stressed plants were grown at 20 °C throughout. Bars represent standard deviations. Means with the same letter are not statistically significantly different, following Tukey's HSD test. Sample size varied from n = 8 to n = 22 with an average of n = 12.

Supplementary Table 1. Pollen viability in the flowers of plants exposed to different temperatures.

Accession	20 °C	23 °C	26 °C	28 °C<br>%	30 °C	32 °C	34 °C
Spring 32 (WT)	95.4 ± 3.3[a]	91.2 ± 5.6[ab]	61.6 ± 5.1[a]	36.6 ± 3.8[a]	14.2 ± 3.8[a]	7.2 ± 3.8[a]	5.4 ± 4.0
rod1 (+T)	91.9 ± 3.7[a-c]	89.1 ± 3.2[ab]	77.7 ± 3.4[b]	55.4 ± 3.8[b]	34.6 ± 4.8[b]	17.9 ± 5.0[bc]	7.4 ± 3.2
fae1 (-4)	91.9 ± 7.9[a-c]	85.6 ± 3.3[a]	52.5 ± 3.7[a]	33.5 ± 7.3[a]	18.2 ± 4.2[a]	10.1 ± 6.2[ab]	2.5 ± 2.2
fad2 (+A)	84.8 ± 4.2[c]	95.9 ± 2.5[b]	82.8 ± 9.4[b]	71.2 ± 6.4[c]	51.1 + 5.3[c]	20.1 ± 1.9[c]	7.6 ± 4.5
fad2 (-29)	85.4 ± 5.6[bc]	92.8 ± 3.3[b]	81.7 ± 6.5[b]	70.0 ± 3.4[c]	50.3 ± 10.0[c]	19.9 ± 4.9[b]	5.9 ± 3.8
rod1 fae1 (+T, -4)	95.0 ± 5.4[ab]	89.3 + 2.8[ab]	73.9 ± 3.1[b]	51.3 ± 4.0[b]	32.2 ± 6.5[b]	18.8 ± 5.4[bc]	3.3 ± 2.6
fad2 fae1 (+A, -4)	84.1 ± 3.8[bc]	94.4 ± 5.1[b]	85.5 ± 4.4[b]	71.5 ± 8.3[c]	51.8 ± 6.5[c]	19.7 ± 3.4[c]	5.2 ± 3.2

Source of Variation	$F_{6,44}$	$F_{6,44}$	$F_{6,44}$	$F_{6,44}$	$F_{6.44}$	$F_{6,44}$	$F_{6,44}$
Type	6.99***	5.66***	29.64***	71.45***	36.51***	12.26***	2.05
Mutation	0.30	3.36	0.62	0.03	0.03	0.04	0.48

The data is from Experiment 1. Flowering plants were exposed to 7-hour treatments at temperatures ranging from 20 °C to 34 °C, in wild-type *Thlaspi arvense* (Spring 32-10) and six gene-edited lines. Data are from a growth chamber experiment, in which plants had previously been grown, until flowering, at 20 °C. Temperatures were first set to 20 °C, then progressively raised by 2-3 °C each day, and pollen was collected on consecutive days (at the end of the peak temperature period, at the equivalent of 13 hours after dawn). In the analysis of variance, symbols '***' and '****' represent statistical significance at $\alpha = 0.001$ and 0.0001, respectively. "Type" refers to the effect of loss of function of a particular gene, and "mutation" to the specific gene-edited allele. Means with the same letter are not statistically significantly different following Tukey's test. Sample size varied from n = 4 to n = 11 with an average of n = 7.
